# Chemically Advanced Template Search (CATS) for Scaffold-Hopping and Prospective Target Prediction for ‘Orphan’ Molecules

**DOI:** 10.1002/minf.201200141

**Published:** 2013-02-07

**Authors:** Michael Reutlinger, Christian P Koch, Daniel Reker, Nickolay Todoroff, Petra Schneider, Tiago Rodrigues, Gisbert Schneider

**Affiliations:** [a] ETH, Department of Chemistry and Applied Biosciences, Institute of Pharmaceutical SciencesWolfgang-Pauli-Str. 10, CH-8093 Zurich, Switzerland fax: +41 44 633 13 79, tel: +41 44 633 74 38

**Keywords:** Drug design, Similarity searching, Combinatorial chemistry, Orphan drug, Polypharmacology, Self-organizing map, Virtual screening

Drug discovery is driven by the identification of new chemical entities (NCEs).^1^,^2^ Virtual screening and de novo design techniques have been proven to serve this purpose, thereby complementing experimental biochemical and biological approaches.^3^ Still, it remains a matter of debate, which particular molecular representation and similarity index are preferable for a given drug target in order to identify appropriate NCEs with minimal synthetic and testing effort involved.^4^ Ligand-based chemical similarity approaches have been effectively applied to large-scale activity and target prediction for known drugs, some of the prominent methods being PASS developed by Poroikov et al.,^5^ the techniques conceived by Mestres and co-workers,^6^ and the similarity ensemble approach (SEA) implemented by the Shoichet group.^7^ Here, we compared several popular two-dimensional molecular representations for their ability to retrieve actives (enrichment potential) and chemotypes (scaffold-hopping potential) from a collection of druglike bioactive compounds. Subsequently the applied chemical advanced template search (CATS)^8^ was applied to predicting potential drug targets for a virtually assembled combinatorial compound library, from which we synthesized and successfully tested candidate compounds. The results demonstrate that CATS is not only suited for its intended purpose of NCE retrieval by scaffold-hopping,^9^ but also for reliable target profiling of ‘orphan’ virtual molecules.^10^ It thereby complements the suite of available validated tools for target prediction.

A framework for retrospective evaluation of similarity searching runs with different molecular representations (‘descriptors’) was established on basis of the COBRA collection of druglike bioactive compounds,^11^ employing Euclidean distances for metric descriptors and the Tanimoto coefficient for fingerprint descriptors.^12^ COBRA contains 12 642 manually curated entries with 980 target protein subtype annotations. For 170 macromolecular drug targets with a minimum of 20 annotated active ligands per target, each compound annotated as ‘active’ was selected as a query in turn, and compared to all remaining compounds in the screening pool in terms of molecular descriptor similarity, finally yielding sorted results lists with the most similar or least distant pool compounds sorted to the top. Although there are large collections of bioactive compounds available in the public domain,^13^ we used the carefully compiled COBRA collection to i) reduce the risk of erroneous activity data and faulty compound structures,^14^ and ii) avoid redundancy with existing tools that are based on such public structure-activity data. In addition, we intend to probe the value of a comparably small but well curated reference compound pool for target prediction.

We used a representative set of descriptors and fingerprints for benchmarking. ‘Morgan’ fingerprints, closely related to extended-connectivity fingerprints (ECFP), are based on radial assessment of non-predefined potentially infinite molecular fragments.^17^ The ‘AtomPair’ descriptor can be seen as a CATS predecessor merely denoting the occurrence of all pairs of atoms at a given topological distance.^18^ The ‘MACCS’ keys represent substructure-based fingerprints,^19^ and the ‘RDkit’ fingerprint implements a Daylight-like fingerprint based on hashed molecular subgraphs.^20^ Latter fingerprints and descriptors were calculated using the open-source software package RDkit.^21^ Finally, the ‘MOE2D’ descriptor consists of a standardized vector of physicochemical properties provided by the Molecular Operating Environment (v2011, Chemical Computing Group, Montreal).

At this point, we analyzed two versions of CATS vectors, namely the originally described CATS1^8^ and CATS2, which distinguishes lipophilic from aromatic atoms during typing, thereby resulting in more pharmacophore type pairs and consequently a higher dimensionality of the descriptor than CATS1, which lacks the aromatic atom type. For both descriptors we employed ‘types scaling’, which mitigates the potential dominance of prevalent pharmacophore feature types, and a maximal correlation distance of 10 bonds.^22^ An example of CATS descriptor calculation is presented in Figure 1.

**Figure 1:**
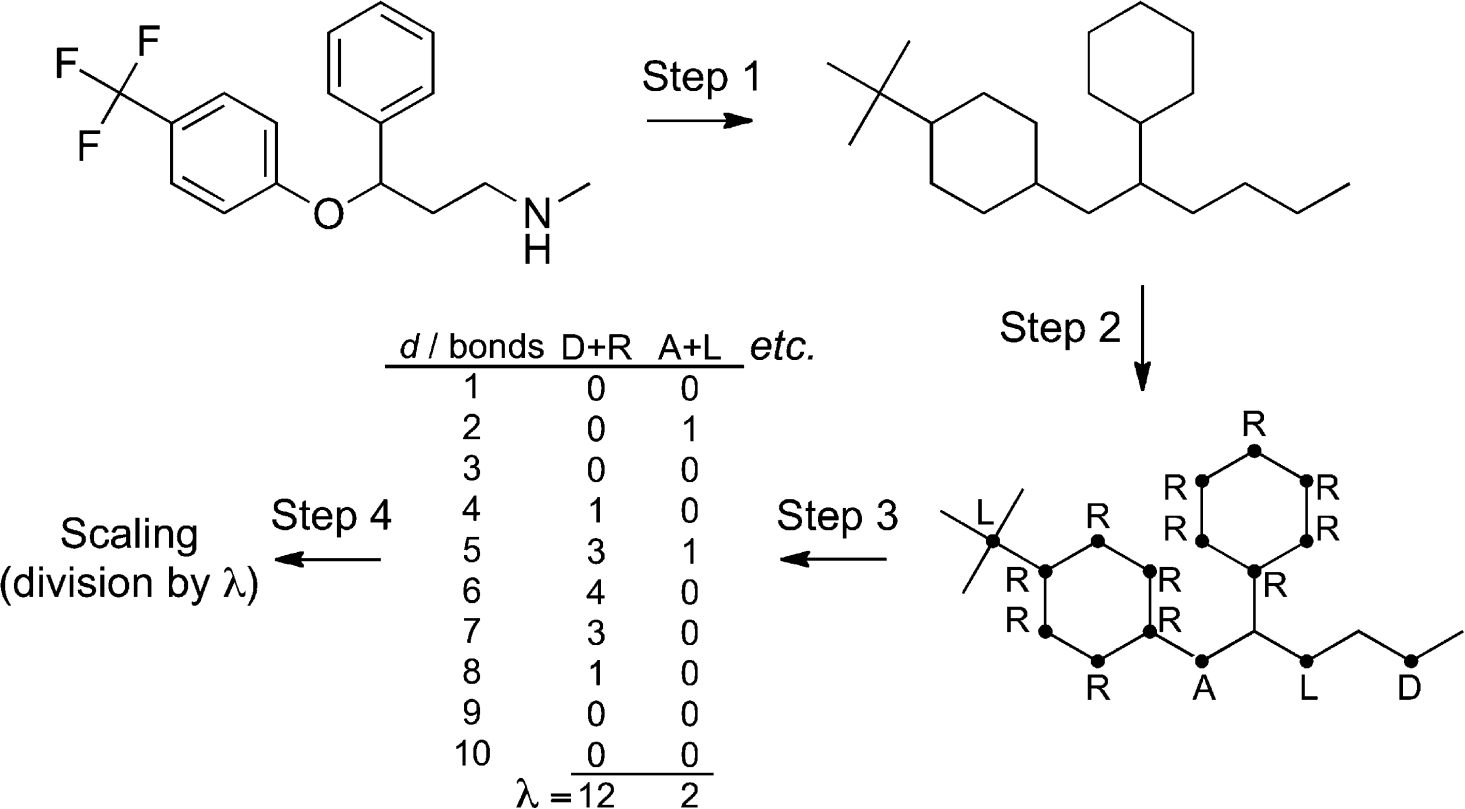
Principle of CATS descriptor calculation. The molecular structure (Step 1) is reduced to the molecular graph, and feature types are assigned (Step 2; L, lipophilic; R, aromatic; A, hydrogen-bond acceptor; D, hydrogen-bond donor). Then, atom pairs for all feature pairs are counted (Step 3), and the final descriptor values are scaled (Step 4). Here, the raw values were divided by the respective λ value (sum of atom type pair occurrences). Note that not all vertices in the molecular graph are considered ‘pharmacophoric’. These possess no feature types.

We employed the Receiver Operating Characteristic (ROC) related BEDROC score for actives-retrieval benchmarking.^15^ For our study, the alpha level of the BEDROC method was set to 160.9, which corresponds to the top 1 % of the screening list contributing 80 % of the score. Murcko scaffold^16^ diversity among the set of actives within the top 1 % of respective screening lists served as measure for scaffold-hopping potential.

Albeit state-of-the-art radial fingerprints and atom-pair fingerprints outperformed CATS descriptors in terms of the number of actives retrieved (Figure 2A), the latter ratify their intent of design by delivering the overall highest ratio of diverse scaffolds among retrieved actives. Scaffold-hopping potential was determined by examining the distribution of relative scaffold diversities *r*, which is the ratio of differing scaffolds *s* to the number of retrieved actives *n* among the top 1 % of respective screening runs. While *s* correlates to the BEDROC scores when comparing different descriptors, *r* unveils the CATS1 descriptor as the most suitable descriptor for scaffold-hopping among the compared molecular representations (Figure 2B). In terms of BEDROC scores estimating the enrichment potential, radial fingerprints (Morgan) and Carhart-type atom pairs (AtomPair) performed similar, as did the CATS2 and MOE descriptors, while MACCS, CATS1, and RDkit fingerprints formed a third group (Figure 3A). With respect to scaffold-hopping potential, the groups vary, with CATS1 and MOE2D pairing up, as well as CATS2 and MACCS (Figure 3B). It might thus be advisable to select one method from each group for similarity searching and compare ranked results lists, *e.g.* by data fusion.^23^ We wish to point out that the grouping of methods depicted in Figure 3 should be treated with caution, as the dendrograms are likely to vary for other reference data sets and chemotype/target coverage.

**Figure 2:**
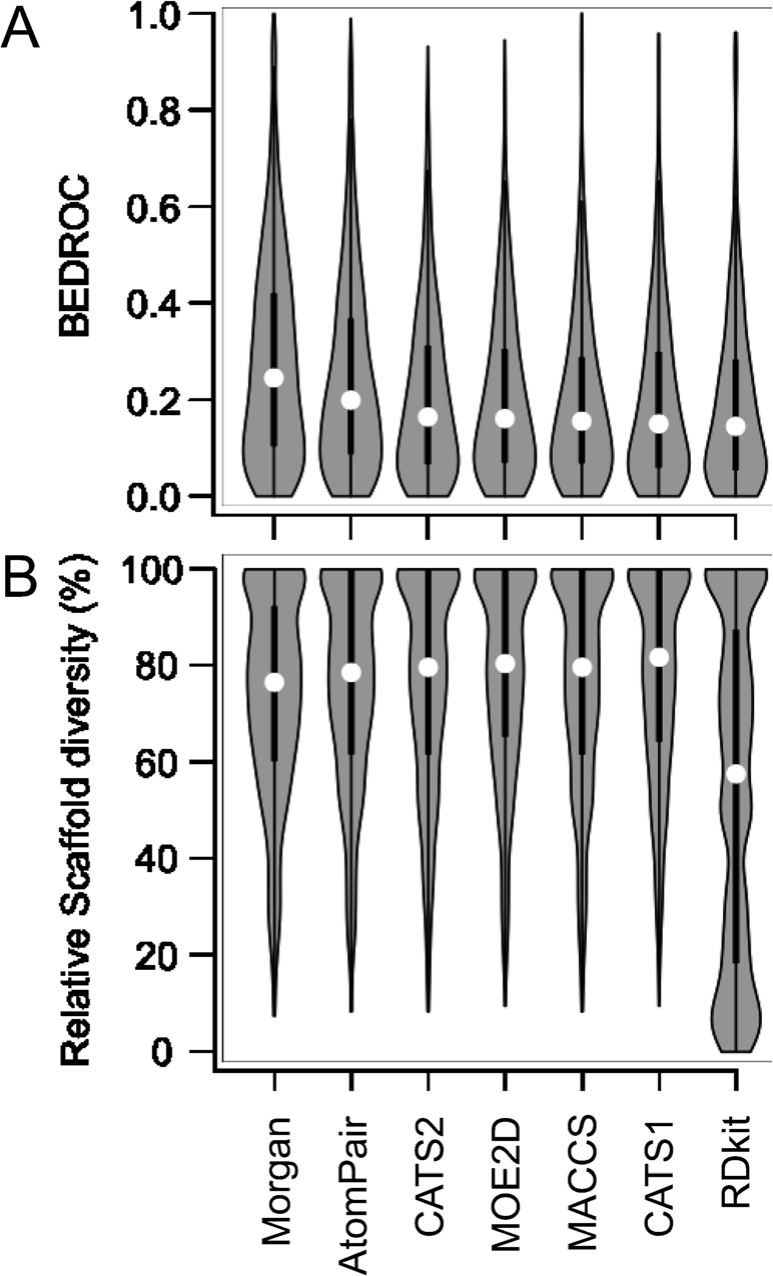
Comparison of molecular representations for their abilities to retrieve known actives (A) and scaffolds (B) from a collection of druglike bioactive compounds (COBRA). Violin plots show the shapes (gray), medians (white circle) and quartiles (thick lines) of the distributions.

**Figure 3:**
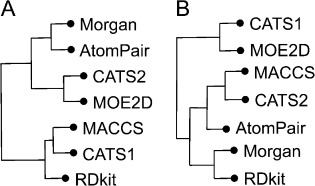
Similarity of molecular representations in terms of their enrichment (A) and scaffold-hopping potential (B). Pair-wise, one-sided Wilcoxon rank sum tests^24^ were performed for the BEDROC score distributions of the descriptors. Clustering the obtained *p*-values with Ward’s method^25^ resulted in the depicted dendrograms.

The outcome of this limited benchmark study is in agreement with a large-scale systematic analysis of 2D fingerprint methods by Sherman and co-workers, who conclude (…) *if the objective of a screen is to identify novel, diverse hits, then a less specific atom-typing scheme may be more appropriate*.^26^ The CATS representation of molecular graphs and pharmacophoric features serves this purpose of finding new chemotypes. When using the descriptor, one should not expect highest possible enrichment of actives among the top-scoring virtual hits, but can anticipate surprising new ideas for synthesis and activity testing.

This intended permissiveness (‘fuzziness’)^27^ of the CATS molecular representation, which is achieved by coarse-grained atom-typing and feature pair correlation, not only enables scaffold-hopping but may also be used for predicting mutual targets of structurally diverse bioactive ligands. Here, we started from an Ugi-type three-component combinatorial synthesis (Scheme 1)^28^ and tested whether we could use CATS for ‘de-orphanizing’ some of the compounds by target identification. All prospective experiments were carried out with the CATS2 implementation.

**Scheme 1:**
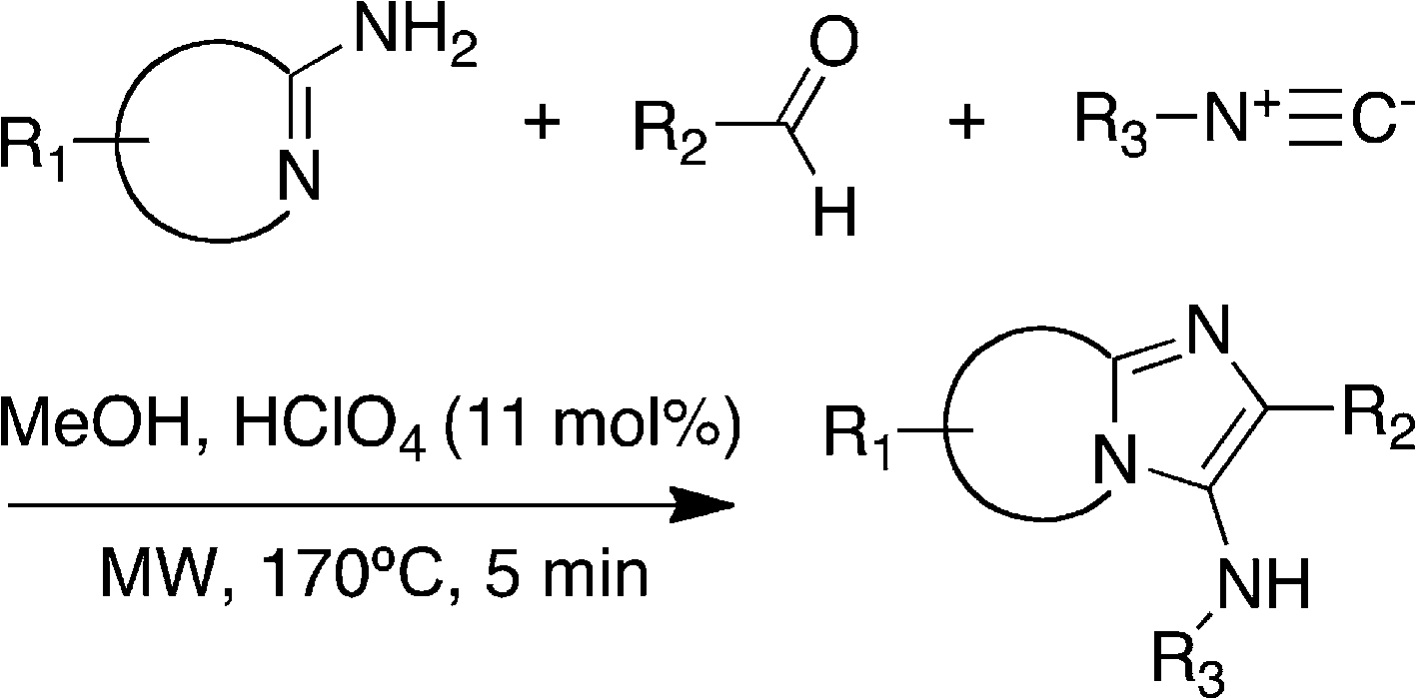
Ugi-type three-component reaction used for constructing a virtual combinatorial library and synthesizing selected compounds.

We constructed a virtual combinatorial library from 12 aminopyridines, 40 aldehydes and 8 isocyanide building blocks, resulting in 3840 virtual products (Scheme 1 and Supporting Information). To predict potential bioactivities for these compounds we computed their CATS similarity values to known drugs and lead structures (COBRA v11.10). Briefly, we trained a self-organizing neural network (SOM, Kohonen network) on the pool of COBRA reference compounds and the virtual combinatorial products, followed by visualization of compound distributions as a two-dimensional toroidal map (Figure 4).^29^,^30^ For the purpose of prediction, we only considered annotated targets of the reference compounds that were co-clustered with the combinatorial products. In this way, target predictions are limited to a conservative ‘application domain’ of a reference compound cluster, and the risk of false-positive prediction is reduced.^31^ For further target prioritization, we computed *p*-values from the similarity score distribution between ligands binding to different targets (complete training data).^32^ The *p*-values are an estimate of the probability of making a false-positive prediction (type-I error).

**Figure 4:**
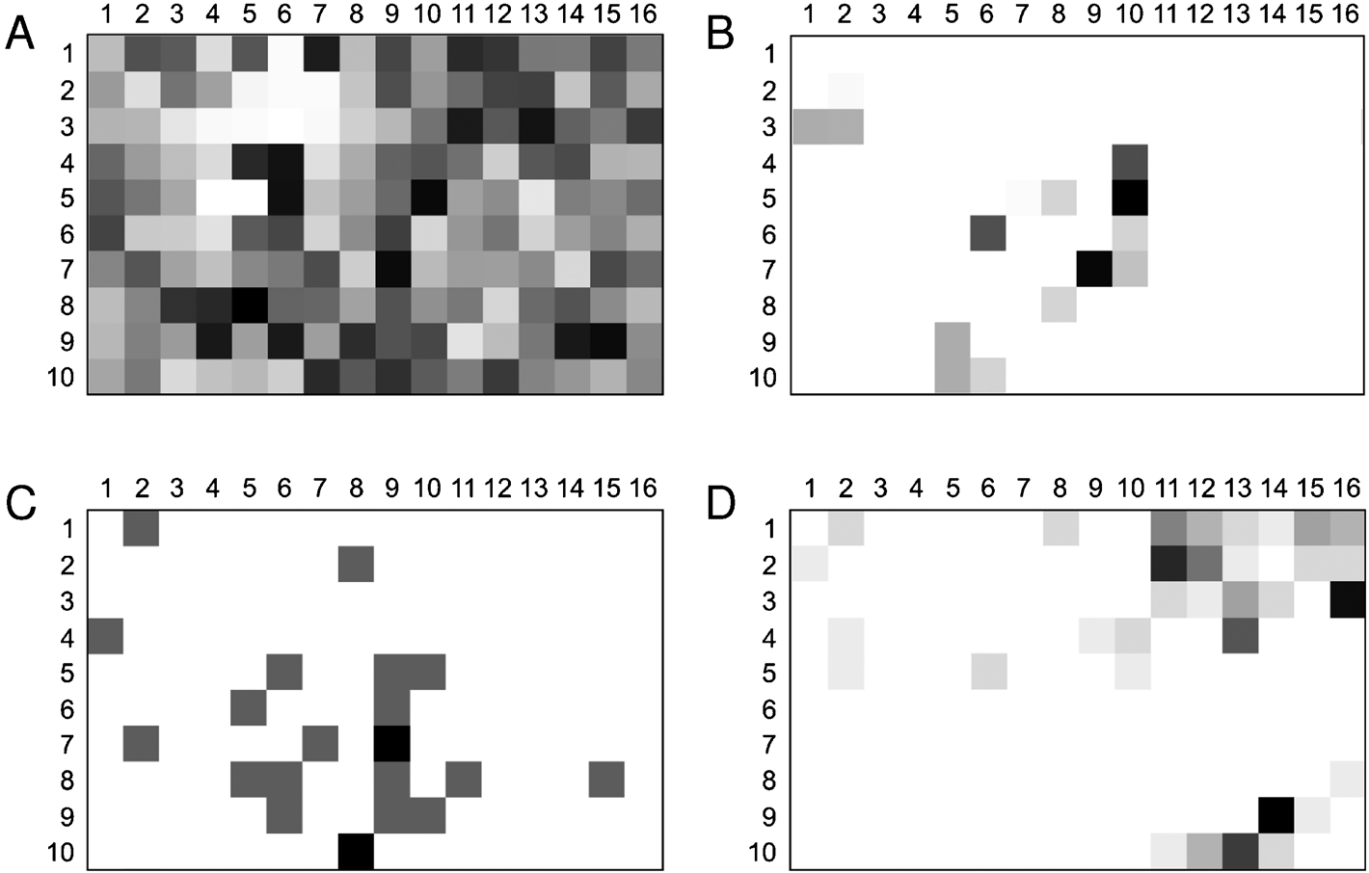
Toroidal self-organizing map (SOM) visualizing overall data density (A), distribution of the virtual combinatorial library (B), known PI3K inhibitors (C), and known muscarinic receptor ligands (D). 16×10 data clusters (‘neurons’, Voronoi fields) are shown as squares. Gray shading represents local compound density (note that the shading in each plot is scaled between minimal and maximal values). Compound **1** is located in cluster (9,7), compound **2** in cluster (10,5). For compound **1** an overlap with PI3K inhibitors is predicted. Compound **2** is found in a cluster that contains muscarinic receptor ligands and few PI3K inhibitors.

For the whole library, this method suggested six targets with average *p*-values <0.01: phosphoinositide 3-kinase (PI3K), biphenyl-2,3-diol 1,2-dioxygenase, diacylglyceride *O*-acyltransferase, smoothened receptor, interleukin receptors, and cytochrome P450 reductase. We decided to investigate the PI3K prediction in more detail because this enzyme is a relevant drug target in antitumor research. Of note, the underlying scaffold was previously shown to afford PI3Kα inhibitors.^33^

First, we synthesized and tested the nine top-predicted compounds for PI3Kα inhibition. In total, four of them exhibited the desired activity. Compound **1** (Scheme 2) turned out to be the most active (*IC*_50_=131 µM). Although the measured activities might be considered as weak, this result nevertheless proves the CATS+SOM-based approach valid for suggesting plausible macromolecular targets for small molecules.

**Scheme 2:**
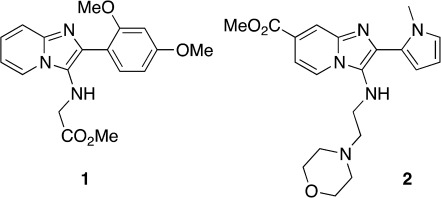
Structures of compounds **1** and **2**.

We then synthesized and tested an additional set of 57 compounds from the virtual combinatorial library, for which the highest joint prediction scores for PI3K and DNA topoisomerases were computed. These were simply the top DNA topoisomerase hits that were also predicted to inhibit PI3K with relatively high confidence. Previous studies suggested that simultaneous inhibition of these two enzymes might allow for more efficient chemotherapy with reduced chemoresistance of tumor cells.^34^ Molecules with a target profile that includes both these targets will constitute an important step in anti-cancer research. Moreover, the scaffold of our library has already been proven to produce bioactive compounds against both those targets.^33^, ^35^ In fact, in the present study six of our compounds, at a concentration of 75 µM, turned out to be moderately active against PI3Kα, where compound **2** (Scheme 2) was the most potent (*IC*_50_=230±30 µM). We wish to point out that we cannot completely rule out measurement artifacts caused by compound aggregation.^36^ None of the 57 synthesized compounds inhibited human DNA topoisomerse II (EC 5.99.1.3), but in a preliminary test four of them inhibited bacterial DNA gyrase, a bacterial type II topoisomerase (EC 5.99.1.3) (data not shown). Apparently, the scaffold of the combinatorial library positions R-group vectors appropriately, but proper side-chain functionalities are required for potency and target selectivity. There is ample opportunity for optimizing compound **2** in this regard by including additional building blocks in the combinatorial synthesis.

For comparison, we also predicted targets for the obtained PI3Kα inhibitors using SEA^39^. In SEA, compound **2** yielded no target predictions at all when using ChEMBL^40^ as reference data. For the remaining compounds SEA reported maximal Tanimoto similarity below 0.35 and *E*-value>1.2, rendering them low confidence predictions. Compound **1** was suggested as ligand of quinone reductase 2 (NQO2) and melatonin receptor 1B (MTNR1B). PI3K was not reported as a potential target for compound **1** by SEA.

Finally, it is of particular note that CATS suggested human muscarinic receptor 1 (M1) ranking among the top predictiones on the target list computed just for compound **2**. In a first cell-based functional assay^[41]^ compound **2**, in a concentration of 10 µM, actually exhibited substantial M1 agonistic activity yielding 34±5 % of the effect caused by 100 nM acetylcholine. Follow-up concentration-dependent activity determination yielded an approximate *EC*_50_ of 5 µM for compound **2** (Figure 5). This result confirms the CATS+SOM-based target prediction as viable and de-orphanizes compound **2** as a novel (no entry in CAS^42^) functional M1 receptor agonist. We would like to mention that the SOM projection shown in Figure 4D may actually serve as a guide for structure optimization,^29^,^37^ due to the fact that compound **2** is located in a sparsely populated region of the activity island formed by known muscarinic receptor ligands. Side-chain alteration could steer the design towards the center of the distribution thus potentially improving potency.^38^

**Figure 5:**
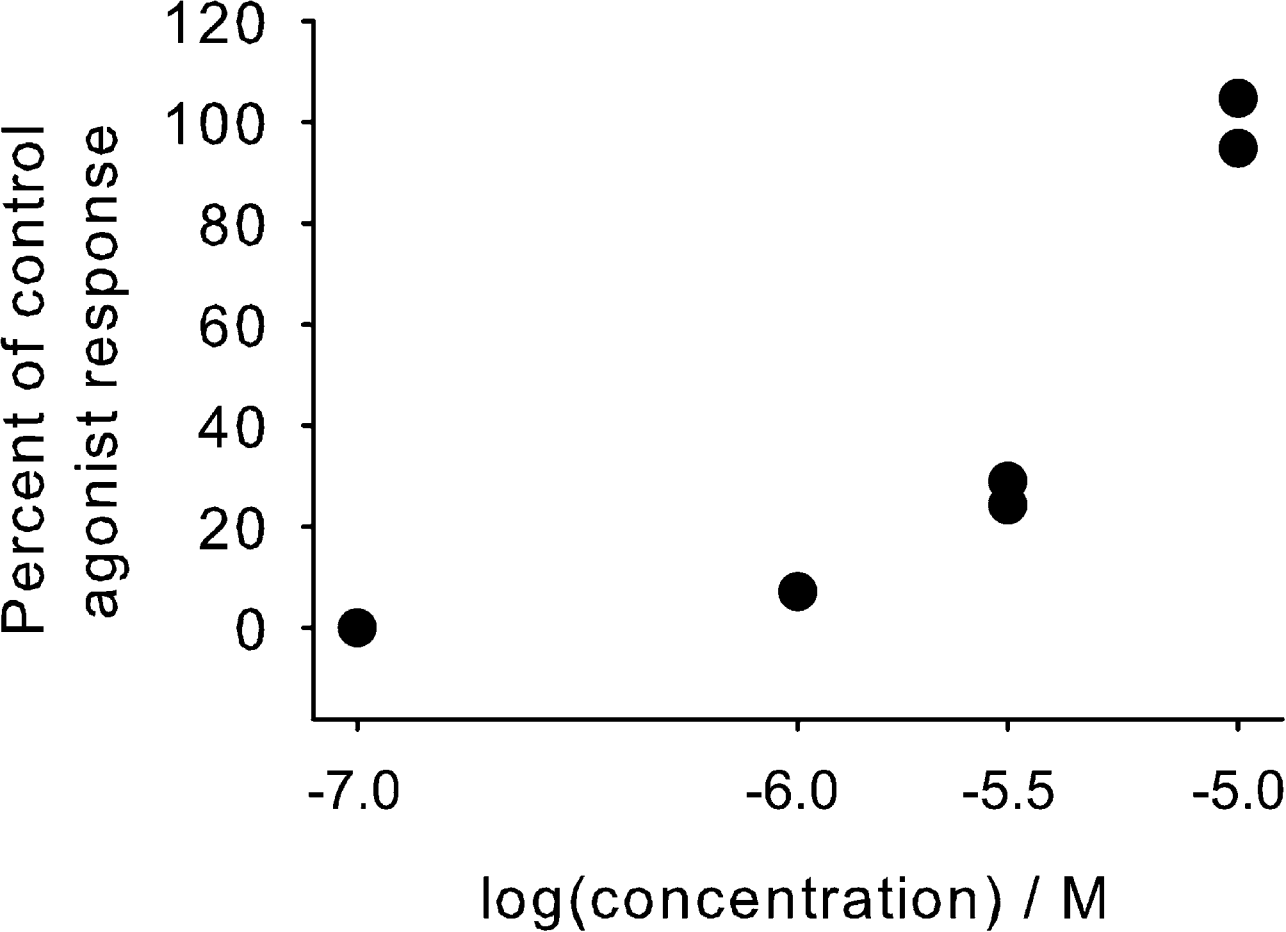
Concentration-dependent agonistic activity of compound **2** on the human M1 receptor. Acetylcholine served as positive control agonist (*EC*_50_=1.9 nM). At ligand concentrations>10 µM compound **2** aggregated and interfered with the measurement (data not shown).

In conclusion the results of this study corroborate CATS+SOM as a useful similarity-based approach for identifying pairs of molecules with similar bioactivity but different molecular scaffolds. Inclusion of the aromatic feature type in the CATS2 implementation increased enrichment in a retrospective analysis. Results of a preliminary prospective target-profiling study demonstrate that (i) the CATS2 descriptor may be employed to predict targets of virtually generated compounds with potential applications in de novo design and drug re-purposing, (ii) relying only on a single prediction algorithm bears the danger of missing relevant drug targets or focusing on false-positive predictions, and (iii) different molecular descriptors (here: CATS2; SEA with ECFP4 fingerprints) in combination with its associated knowledge base (here: COBRA or ChEMBL) complement each other in their domains of applicability. It will therefore be worthwhile to construct a prediction tool that is based on multiple reference databases, descriptors and models, *e.g.* as a jury decision approach. Whether activities in the micromolar range give rise to desired poly-pharmacology effects or turn out to be actually sufficient for drug re-purposing certainly depends on the particular pharmacological activity, therapeutic area, and intended application.^43^ Many more practical examples will be required to allow for a statistically motivated assessment. Irrespective of the shortcomings of each method, our study validates ligand-based target prediction as viable for rapid compound profiling in medicinal chemistry and chemical biology.

## Experimental

*Synthesis and analytics*. Chemical synthesis was performed with a Biotage Initiator microwave synthesizer (Upsala, Sweden). Aminopyridine (1.0 mol. eq.), aldehyde (1.0 mol. eq.), isocyanide (1.0 mol. eq.) and perchloric acid (11 mol%) were dissolved in EtOH (1.1 mL×mmol^−1^). The solution was heated at 170 °C for 5 minutes under microwave irradiation. The resulting crude product was purified via preparative HPLC using CH_3_CN:H_2_O (+0.1 % trifluoroacetic acid in each phase) as eluent, in a gradient of 5–50 % CH_3_CN run over 16 minutes, to afford compounds **1** and **2** as yellow oils.

Compound **1** (methyl 2-((2-(2,4-dimethoxyphenyl)imidazo[1,2-*a*]pyridin-3-yl)amino)acetate), 81 %: ^1^H-NMR (CD_3_OD, 400.13 MHz): *δ* 3.44 (3H, s, OCH_3_), 3.67 (2H, s, CH_2_), 3.75 (3H, s, OCH_3_), 3.80 (3H, s, OCH_3_), 6.57–6.61 (2H, m, Ar-*H*), 7.32–7.36 (1H, m, Ar-*H*), 7.53 (1H, d, *J=*8.0 Hz, Ar-*H*), 6.77–7.75 (2H, m, Ar-*H*), 8.68 (1H, d, *J=*2.4 Hz, Ar-*H*). ^13^C NMR (CD_3_OD, 100.61 MHz): *δ* 48.54, 52.52, 56.18, 56.48, 99.77, 107.08, 108.44, 112.51, 117.43, 123.13, 126.42, 129.04, 132.62, 133.56, 137.46, 159.91, 164.60, 173.19. HRMS-ESI calc. (C_18_H_19_N_3_O_4_+H^+^): 342.1448, found: 342.1448.

Compound **2** (methyl 2-(1-methyl-1*H*-pyrrol-2-yl)-3-((2-morpholinoethyl)amino)imidazo[1,2-*a*] pyridine-7-carboxylate), 74 %: ^1^H-NMR (CD_3_OD, 400.13 MHz): *δ* 3.08 (2H, m, C*H*_2_), 3.21 (2H, t, *J=*6.4 Hz, C*H*_2_), 3.37 (2H, m, C*H*_2_), 3.45 (2H, t, *J=*6.4 Hz, C*H*_2_), 3.72 (3H, s, C*H*_3_), 3.81 (2H, m, C*H*_2_), 3.97 (2H, m, C*H*_2_), 4.05 (3H, s, C*H*_3_), 6.32 (1H, dd, *J=*3.8 Hz, Ar-*H*), 6.63 (1H, dd, *J=*3.8 Hz Ar-*H*), 7.06 (1H, m, Ar-*H*), 7.93 (1H, dd, *J=*1.6 and 7.2 Hz, Ar-*H*), 8.40 (1H, m, Ar-*H*), 8.80 (1H, dd, *J=*0.8 and 7.2 Hz, Ar-*H*). ^13^C NMR (CD_3_OD, 100.61 MHz): 35.01, 41.04, 53.33, 53.84, 57.27, 64.81, 110.04, 114.52, 115.63, 116.03, 116.31, 118.78, 126.40, 127.62, 131.28, 134.24, 137.07, 165.19. HRMS-ESI calc. (C_20_H_25_N_5_O_3_+H^+^): 384.2030, found: 384.2031.

We used dynamic light scattering (Brookhaven 90Plus) to determine potential aggregation of compound **2** in aqueous solution with 1 % DMSO. Aggregate particles were observable at concentrations ranging from 15.5–250 µM.

*Self-organizing map*. We use our software tool *molmap* for generating a toroidal SOM containing 160 clusters arranged in a 16×10 rectangular grid, as described previously,^31^ with number of training cycles=10^6^ and Gaussian neighborhood radius=8.

*CATS molecular descriptor*. Descriptor calculation was performed with a proprietary Java-based software tool (for licensing options, contact G. S.). Free online access to demonstration software is provided at URL: http://modlab-cadd.ethz.ch/

*Biochemical activity determination*. Activity against PI3Kα was measured by Reaction Biology Corp. (Malvern, PA, USA) in a 10-dose *IC*_50_ determination (*n*=3), in the presence of 10 µM ATP. Preliminary DNA topoisomerase and gyrase inhibition tests were performed with a compound concentration of 5 mM by Inspiralis Ltd (Norwich, UK).

## Supplementary material

As a service to our authors and readers, this journal provides supporting information supplied by the authors. Such materials are peer reviewed and may be re-organized for online delivery, but are not copy-edited or typeset. Technical support issues arising from supporting information (other than missing files) should be addressed to the authors.
