# Supplementary material for: Chemically Advanced Template Search (CATS) for Scaffold-Hopping and Prospective Target Prediction for ‘Orphan’ Molecules
Source: Mol Inform. 2013 Feb 7;32(2):133–8. doi: 10.1002/minf.201200141 (PMC3743170; doi:10.1002/minf.201200141)
Supplement: Supplementary file 1 [file minf0032-0133-SD1.pdf]

SUPPLEMENTARY INFORMATION

Chemically advanced template search (CATS) for scaffold-hopping and prospective target prediction for 'orphan' molecules

Michael Reutlinger, Christian P. Koch, Daniel Reker, Nickolay Todoroff, Petra Schneider, Tiago Rodrigues, Gisbert Schneider

Table of Contents

General information S2

Virtual combinatorial library – building blocks S3

General procedure for synthesis of imidazopyridine combinatorial library S4

General procedure for synthesis of selected imidazopyridines S4

HPLC chromatograms S10

Preliminary screening data and IC_{50} values (PI3Kα) S29

References S42

General information

All starting materials and solvents were obtained from ABCR Chemicals, Aldrich, Fluka, Alfa Aesar or Acros, and were used without further purification.

Combinatorial library was built using J-KEM® Scientific robot for mixing the building blocks. Microwave-assisted synthesis was then carried out in a Biotage Initiator reactor.

Analytical HPLC-MS was carried out in a Shimadzu LC-MS2020 system, equipped with a Nucleodur C_{18} HTec column, under an appropriate gradient of acetonitrile : H_2O (+ 0.1% trifluoroacetic acid in each phase), and a total flow rate of 0.5 mL/min. The mass spectrometer was operated in positive-ion mode with ESI. Preparative HPLC was carried out on a Shimadzu LC-8A system, coupled to a Nucleodur 100-5 C_{18} HTec column, and a SPD-20A UV/Vis detector.

Proton and carbon nuclear magnetic resonance spectra (^{1}H and ^{13}C NMR, respectively) were recorded on Bruker Avance 400 spectrometer. Chemical shifts (δ) are reported in units of parts per million (ppm) downfield from $SiMe_4$ (δ 0.0) and relative to the respective solvent's peak. Multiplicities are given as: s (singlet), d (doublet), t (triplet), dd (double of doublet) td (triplet of doublet) or m (multiplet). ^{1}H-^{1}H Coupling constants (*J*) are reported in Hertz (Hz).

Virtual combinatorial library – building blocks

A virtual combinatorial library was built from the building blocks described by Burchak *et al.*[1] The structures of aminopyridines (P), aldehydes (A) and isocyanides (I) are given below.

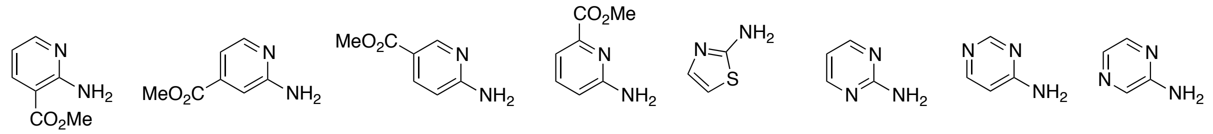

P1 P2 P3 P4 P5 P6 P7 P8

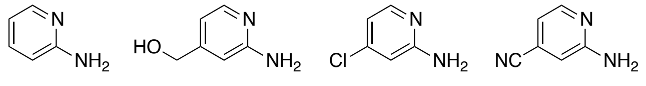

P9 P10 P11 P12

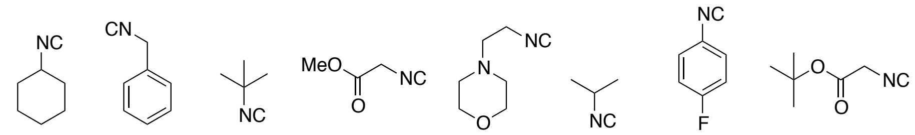

I1 I2 I3 I4 I5 I6 I7 I8

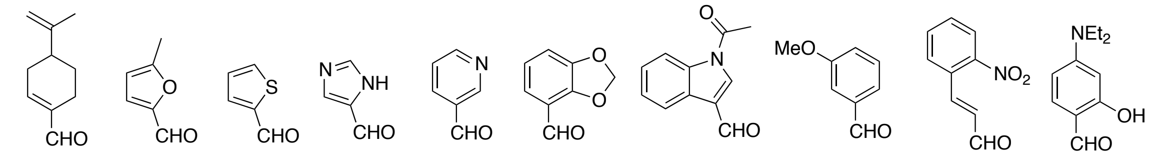

A1 A2 A3 A4 A5 A6 A7 A8 A9 A10

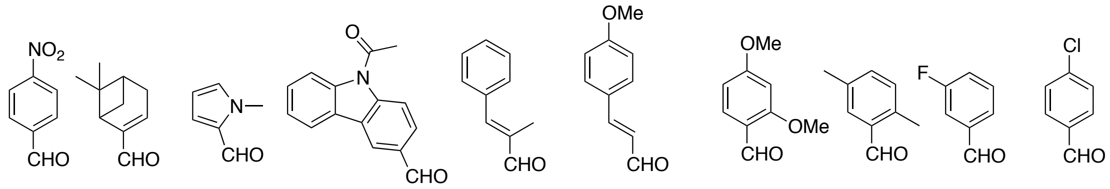

A11 A12 A13 A14 A15 A16 A17 A18 A19 A20

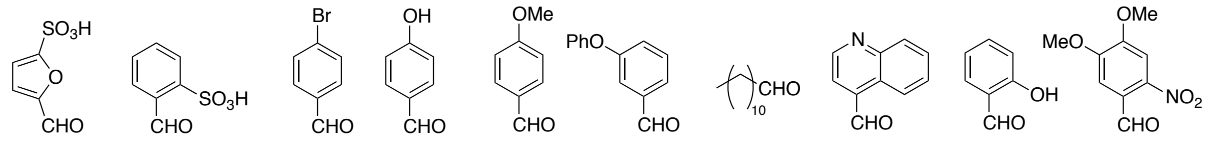

A21 A22 A23 A24 A25 A26 A27 A28 A29 A30

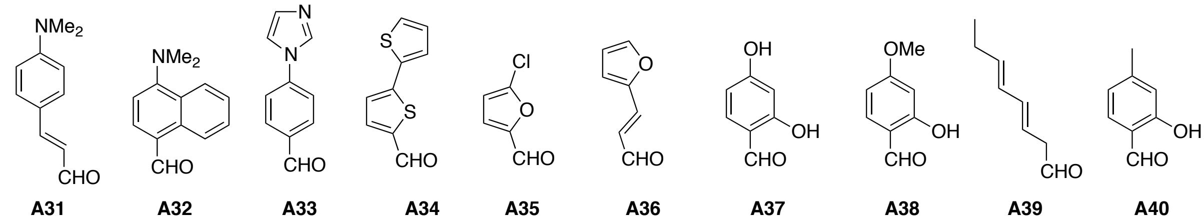

A31 A32 A33 A34 A35 A36 A37 A38 A39 A40

General procedure for synthesis of imidazopyridine combinatorial library

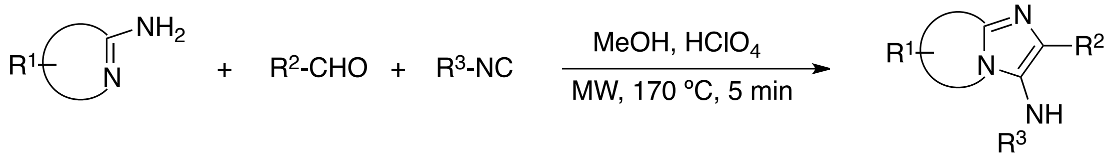

Stock solutions in MeOH were prepared for all building blocks (P, A and I) and catalyst. These solutions had a concentration of 400 mM for P, A and I starting materials, while for perchloric acid a solution of concentration 40 mM was prepared. An automated system dispensed 75 μL of each building block and catalyst, sequentially, into microwave vials. These were sealed and heated for 5 minutes, at 170ºC under microwaves. The reaction products were analyzed under HPLC-MS using ACN : H_2O (+ 0.1% TFA in each phase) as eluent. A typical run used a gradient of 5-50% ACN run in 12 minutes.

General procedure for synthesis of selected imidazopyridines

The protocol was adapted from existing literature.[2] 2-Aminopyridine (1.0 molar eq.), aldehyde (1.0 molar eq.) isocyanide (1.0 molar eq.) and perchloric acid (10 mol%) were dissolved in ethanol absolute (2.1 mL/mmol). The solutions were heated at 170 ºC for 5 minutes under microwaves. The resulting crudes were purified via preparative HPLC using ACN : H_2O (+ 0.1% TFA in each phase) as eluent. A typical run used a gradient of 5-50% ACN run over 16 minutes.

Methyl 2-((2-(2,4-dimethoxyphenyl)imidazo[1,2-*a*]pyridin-3-yl)amino)acetate, 1

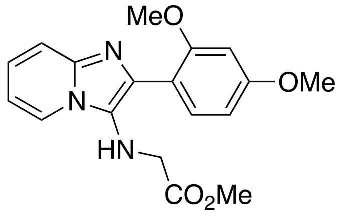

Yellow oil; 81%; ^{1}H NMR (CD_3OD, 400.13 MHz): δ 3.44 (3H, s, OCH_3), 3.67 (2H, s, CH_2), 3.75 (3H, s, OCH_3), 3.80 (3H, s, OCH_3), 6.57-6.61 (2H, m, Ar-H), 7.32-7.36 (1H, m, Ar-H), 7.53 (1H, d, J = 8.0 Hz, Ar-H), 6.77-7.75 (2H, m, Ar-H), 8.68 (1H, d, J = 2.4 Hz, Ar-H). ^{13}C NMR (CD_3OD, 100.61 MHz): δ 48.54, 52.52, 56.18, 56.48, 99.77, 107.08, 108.44, 112.51, 117.43, 123.13, 126.42, 129.04, 132.62, 133.56, 137.46, 159.91, 164.60, 173.19. HRMS-ESI calc. ($C_{18}H_{19}N_3O_4+H^+$): 342.1448, found: 342.1448.

Methyl 2-(1-methyl-1*H*-pyrrol-2-yl)-3-((2-morpholinoethyl)amino)imidazo[1,2-*a*] pyridine-7-carboxylate, 2

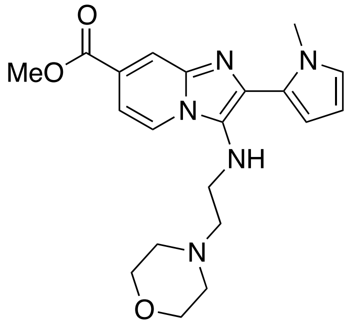

Yellow oil; 74%; (CD_3OD, 400.13 MHz): δ 3.08 (2H, m, CH_2), 3.21 (2H, t, *J* = 6.4 Hz, CH_2), 3.37 (2H, m, CH_2), 3.45 (2H, t, *J* = 6.4 Hz, CH_2), 3.72 (3H, s, CH_3), 3.81 (2H, m, CH_2), 3.97 (2H, m, CH_2), 4.05 (3H, s, CH_3), 6.32 (1H, dd, *J* = 3.8 Hz, Ar-H), 6.63 (1H, dd, *J* = 3.8 Hz Ar-H), 7.06 (1H, m, Ar-H), 7.93 (1H, dd, *J* = 1.6 and 7.2 Hz, Ar-H), 8.40 (1H, m, Ar-H), 8.80 (1H, dd, *J* = 0.8 and 7.2 Hz, Ar-H). ^{13}C NMR (CD_3OD, 100.61 MHz): 35.01, 41.04, 53.33, 53.84, 57.27, 64.81, 110.04, 114.52, 115.63, 116.03, 116.31, 118.78, 126.40, 127.62, 131.28, 134.24, 137.07, 165.19. HRMS-ESI calc. ($C_{20}H_{25}N_5O_3$+H^+): 384.2030, found: 384.2031.

2-(Benzo[*d*][1,3]dioxol-4-yl)-*N*-(2-morpholinoethyl)imidazo[1,2-*a*]pyridin-3-amine, 3

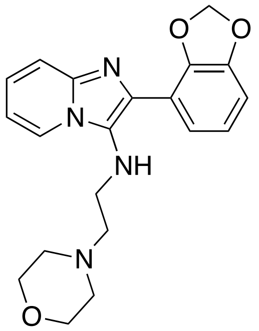

White solid; mp = 131-133 ºC, 75%, ^{1}H NMR (CD_3OD, 400.13 MHz): δ 3.00 (2H, m, CH_2), 3.21 (3H, t, *J* = 6.2 Hz, CH_2 + CH), 3.34 (3H, t, *J* = 6.4 Hz, CH_2 + CH), 3.56-3.94 (4H, m, CH_2), 6.01 (2H, s, CH_2), 6.87 (1H, dd, *J* = 1.8 and 8.2 Hz, Ar-H), 6.92 (1H, t, *J* = 8.2 Hz, Ar-H), 7.14 (1H, dd, *J* = 1.8 and 8.0 Hz, Ar-H), 7.37 (1H, td, *J* = 1.6 and 7.4 Hz, Ar-H), 7.74 (1H, m, Ar-H), 7.82 (1H, m, Ar-H), 8.65 (1H, m, Ar-H). ^{13}C NMR (CD_3OD, 100.61 MHz): δ 42.50, 53.47, 57.66, 64.84, 103.47, 109.52, 111.50, 112.97, 118.24, 122.26, 122.86, 124.07, 126.52, 128.89, 134.85, 138.74, 146.90, 149.91. HRMS-ESI calc. ($C_{20}H_{22}N_4O_3$+H^+): 367.1765, found: 367.1765.

Methyl 2-(2,4-dimethoxyphenyl)-3-((2-morpholinoethyl)amino)imidazo[1,2-*a*]pyridine-6-carboxylate, 4

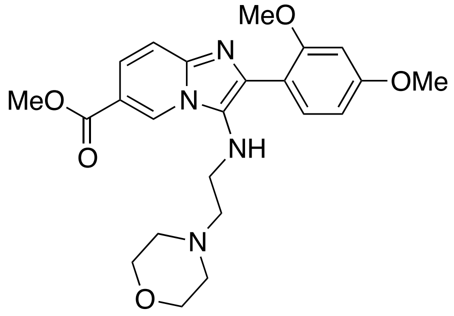

Yellow oil; 92%; ^{1}H NMR ($CDCl_3$, 400.13 MHz): δ 2.90 (2H, m, CH_2), 3.22 (2H, t, *J* = 6.4 Hz, CH_2), 3.32 (2H, t, *J* = 6.4 Hz, CH_2), 3.39 (2H, d, *J* = 11.6 Hz, CH_2), 3.77 (3H, s, OCH_3), 3.82 (3H, s, OCH_3), 3.84-4.00 (4H, m, CH_2), 4.04 (3H, s, OCH_3), 6.42 (1H, d, *J* = 2.0 Hz, Ar-H), 6.45 (1H, dd, *J* = 2.0 and 8.7 Hz, Ar-H), 7.54 (1H, d, *J* = 8.8 Hz, Ar-H), 7.83 (1H, dd, *J* = 1.0 and 9.2 Hz, Ar-H), 8.20 (1H, dd, *J* = 1.6 and 9.4 Hz, Ar-H), 9.11 (1H, s, Ar-H). ^{13}C NMR (CD_3OD, 100.61 MHz): δ 41.89, 53.36, 53.59,

56.22, 56.52, 57.46, 64.79, 99.86, 107.21, 107.91, 112.67, 116.07, 121.89, 125.05, 129.26, 132.88, 133.31, 138.60, 160.23, 165.11, 165.15. HRMS-ESI calc. ($C_{23}H_{28}N_4O_5+H^+$): 441.2132, found: 441.2127.

Methyl 2-(3-fluorophenyl)-3-((2-morpholinoethyl)amino)imidazo[1,2-*a*]pyridine-7-carboxylate, 5

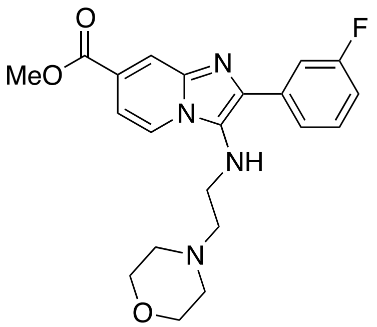

Yellow oil; 70%; ^{1}H NMR ($CDCl_3$, 400.13 MHz): δ 2.96 (2H, m, CH_2), 3.33 (2H, t, J = 6.4 Hz, CH_2), 3.44 (2H, t, J = 6.4 Hz, CH_2), 3.44-3.49 (2H, m, CH_2), 3.90-4.01 (4H, m, CH_2), 4.03 (3H, s, OCH_3), 7.11 (1H, td, J = 2.2 and 8.4 Hz, Ar-H), 7.45 (1H, m, Ar-H), 7.51 (1H, m, Ar-H), 7.59 (1H, d, J = 8.2 Hz, Ar-H), 7.88 (1H, dd, J = 1.6 and 7.0 Hz, Ar-H), 8.40 (1H, s, Ar-H), 8.71 (1H, dd, J = 1.0 and 7.0 Hz, Ar-H). ^{13}C NMR (CD_3OD, 100.61 MHz): δ 42.56, 53.46, 53.86, 57.64, 64.83, 115.00, 116.12, 116.36, 116.62, 118.40, 118.61, 125.44, 126.73, 129.57, 132.77, 134.75, 138.17, 164.52, 165.21. HRMS-ESI calc. ($C_{21}H_{23}FN_4O_3+H^+$): 399.1827, found: 399.1828.

Methyl 2-(3-methoxyphenyl)-3-((2-morpholinoethyl)amino)imidazo[1,2-*a*]pyridine-7-carboxylate, 6

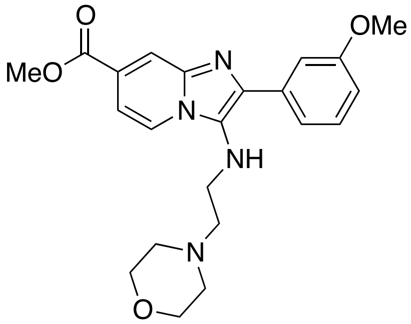

Yellow oil; 93%; ^{1}H NMR ($CDCl_3$, 400.13 MHz): δ 2.88 (2H, m, CH_2), 3.22 (2H, t, J = 6.4 Hz, CH_2), 3.29 (2H, t, J = 6.4 Hz, CH_2), 3.37 (2H, m, CH_2), 3.68 (3H, s, CH_3), 3.82-3.91 (4H, m, CH_2), 3.95 (3H, s, OCH_3), 6.66 (1H, dd, J = 1.6 and 8.2 Hz, Ar-H), 7.06-7.20 (3H, m, Ar-H), 7.72 (1H, dd, J = 1.4 and 7.2 Hz, Ar-H), 8.16 (1H, s Ar-H), 8.55 (1H, d, J = 7.2 Hz, Ar-H). ^{13}C NMR ($CDCl_3$, 100.61 MHz): 41.35, 52.70, 53.52, 55.36, 57.41, 63.53, 112.62, 114.04, 116.17, 116.82, 119.82, 124.39, 126.43, 126.71, 129.48, 130.40, 133.38, 160.18, 161.46, 163.30. HRMS-ESI calc. ($C_{22}H_{26}N_4O_4+H^+$): 411.2027, found: 411.2027.

Methyl 2-(4-methoxyphenyl)-3-((2-morpholinoethyl)amino)imidazo[1,2-*a*]pyridine-8-carboxylate, 7

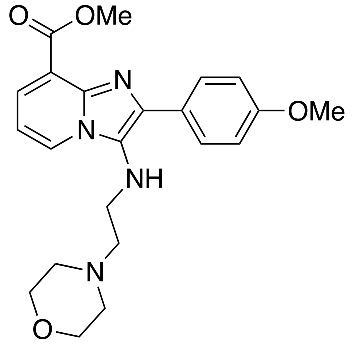

Yellow oil; 72%; ^{1}H NMR ($CDCl_3$, 400.13 MHz): δ 2.88 (2H, m, CH_2), 3.20 (2H, t, *J* = 6.4 Hz, CH_2), 3.37-3.42 (4H, m, CH_2), 3.81 (3H, s, OCH_3), 3.82-4.03 (4H, m, CH_2), 4.03 (3H, s, OCH_3), 6.98 (2H, d, *J* = 9.0 Hz, Ar-H), 7.46 (1H, dd, *J* = 7.2 Hz Ar-H), 7.60 (2H, d, *J* = 9.0 Hz, Ar-H), 8.38 (1H, dd, *J* = 1.0 and 7.4 Hz, Ar-H), 7.98 (1H, dd, *J* = 1.0 and 7.0 Hz, Ar-H). ^{13}C NMR (CD_3OD, 100.61 MHz): δ 42.46, 53.37, 53.86, 56.07, 57.63, 64.79, 115.73, 116.53, 117.69, 119.14, 128.30, 129.59, 130.70 132.39, 136.29, 137.34, 163.12, 164.19. HRMS-ESI calc. ($C_{22}H_{26}N_4O_4$+H^+): 411.2027, found: 411.2028.

Methyl 2-((7-cyano-2-(2,4-dimethoxyphenyl)imidazo[1,2-*a*]pyridin-3-yl)amino)acetate, 8

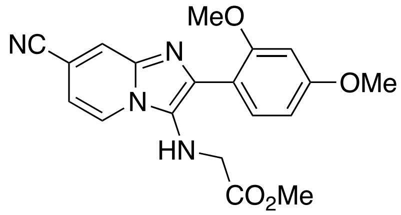

Yellow solid; mp = 60-61 ºC; 87%; ^{1}H NMR (CD_3OD, 400.13 MHz): δ 3.55 (3H, s, OCH_3), 3.80 (2H, s, CH_2), 3.90 (3H, s, OCH_3), 3.92 (3H, s, OCH_3), 6.73 (1H, d, *J* = 2.4 Hz, Ar-H), 6.76 (1H, dd, *J* = 4.8 and 2.0 Hz, Ar-H), 7.62-7.68 (2H, m, Ar-H), 8.28 (1H, s, Ar-H), 8.89 (1H, d, *J* = 7.2 Hz, Ar-H). ^{13}C NMR (CD_3OD, 100.61 MHz): δ 48.17, 52.56, 56.22, 56.51, 99.83, 107.29, 108.31, 114.51, 117.25, 117.60, 118.58, 126.04, 127.11, 131.05, 132.79, 135.66, 160.07, 165.05, 173.04. HRMS-ESI calc. ($C_{19}H_{18}N_4O_4$+H^+): 367.1401, found: 367.1401.

Methyl 2-((2-(benzo[*d*][1,3]dioxol-4-yl)-7-cyanoimidazo[1,2-*a*]pyridin-3-yl)amino) acetate, 9

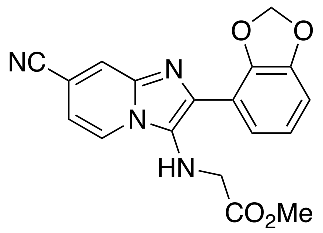

Yellow solid; mp = 160-162 ºC; 56%; ^{1}H NMR (DMSO-d_6, 400.13 MHz): δ 3.49 (3H, s, OCH_3), 3.84 (2H, d, *J* = 6.4 Hz, CH_2), 5.61 (1H, t, *J* = 6.4 Hz, NH), 6.10 (2H, s, CH_2), 6.95-6.97 (2H, m, Ar-H), 7.20 (1H, d, *J* = 7.2 Hz, Ar-H), 7.31 (1H, dd, *J* = 4.4 Hz, Ar-H), 8.23 (1H, s, Ar-H), 8.47 (1H, d, *J* = 6.8 Hz, Ar-H). ^{13}C NMR (DMSO-d_6, 100.61 MHz): δ 47.53, 51.57, 100.83, 103.97, 107.98, 111.44, 115.88, 118.44, 121.73 121.77, 123.34, 124.10, 129.74, 131.54, 138.01, 144.21, 147.36, 171.58. HRMS-ESI calc. ($C_{18}H_{14}N_4O_4$+H^+): 351.1088, found: 351.1087.

Methyl 2-((2-(4-methoxyphenyl)imidazo[1,2-*a*]pyrazin-3-yl)amino)acetate, 10

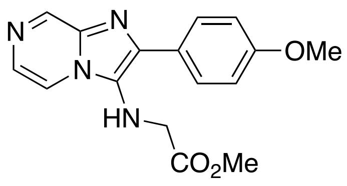

Yellow solid; mp = 150-151 ºC; 67%; ^{1}H NMR (CD_3OD, 400.13 MHz): δ 3.50 (3H, s, OCH_3), 3.78 (3H, s, OCH_3), 3.87 (2H, s, CH_2), 7.00 (2H, d, *J* = 6.8 Hz, Ar-H), 7.83-7.87 (3H, m, Ar-H), 8.56 (1H, d, *J* = 4.8 Hz, Ar-H), 8.92 (1H, s, Ar-H). ^{13}C NMR (CD_3OD, 100.61 MHz): δ 48.06, 52.67, 55.96, 115.55, 118.72, 123.80, 124.50, 130.54, 133.56, 134.82, 135.96, 141.33, 162.58, 173.27. HRMS-ESI calc. ($C_{16}H_{16}N_4O_3$+H^+): 313.1295, found: 313.1295.

Methyl 2-((7-cyano-2-(pyridin-3-yl)imidazo[1,2-*a*]pyridin-3-yl)amino)acetate, 11

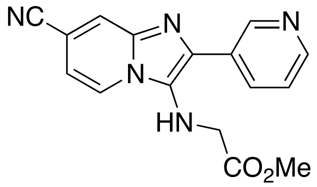

Yellow solid; mp = 183-185 ºC; 39%; ^{1}H NMR (CD_3OD, 400.13 MHz): δ 3.54 (3H, s, CH_3), 3.93 (2H, s, CH_2), 7.28 (1H, dd, *J* = 7.2 and 2.0 Hz, Ar-H), 7.84 (1H, ddd, *J* = 8.0, 5.6 and 0.4 Hz, Ar-H), 8.29 (1H, dd, *J* = 1.6 and 1.2 Hz, Ar-H), 8.63 (1H, dd, *J* = 7.2 and 0.8 Hz, Ar-H), 8.71 (1H, dd, *J* = 5.2 and 0.8 Hz, Ar-H), 8.80 (1H, dt, *J* = 8.0 and 2.0 Hz, Ar-H), 9.40 (1H, d, *J* = 2.0 Hz, Ar-H). ^{13}C NMR (CD_3OD, 100.61 MHz): δ 47.93, 51.65, 105.81, 111.63, 118.09, 123.53, 125.27, 125.42, 130.11, 130.94, 131.82, 137.72, 138.61, 143.68, 144.48, 171.97. HRMS-ESI calc. ($C_{16}H_{13}N_5O_2$+H^+): 308.1142, found: 308.1143.

Methyl 2-((2-(benzo[*d*][1,3]dioxol-4-yl)imidazo[1,2-*a*]pyridin-3-yl)amino)acetate, 12

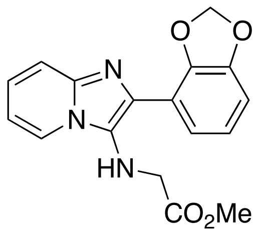

Yellow oil; 80%; ^{1}H NMR (CD_3OD, 400.13 MHz): δ 3.36 (3H, s, OCH_3), 3.68 (2H, s, CH_2), 5.93 (2H, s, CH_2), 6.76 (1H, d, *J* = 8.0 Hz, Ar-H), 6.82 (1H, t, *J* = 8.0 Hz, Ar-H), 7.10 (1H, d, *J* = 8.0 Hz, Ar-H), 7.29 (1H, t, *J* = 7.2 Hz, Ar-H), 7.65 (1H, d, *J* = 9.2 Hz, Ar-H), 7.72 (1H, m, Ar-H), 8.63 (1H, d, *J* = 6.8 Hz, Ar-H). ^{13}C NMR (CD_3OD, 100.61 MHz): δ 48.84, 52.57, 103.33, 109.86, 111.18, 112.83, 117.87, 121.63, 123.98, 126.80, 129.62, 134.50, 134.57, 138.48, 146.54, 149.77, 173.17. HRMS-ESI calc. ($C_{17}H_{15}N_3O_4$+H^+): 326.1135, found: 326.1133.

Methyl 2-((2-(3-methoxyphenyl)imidazo[1,2-*a*]pyrazin-3-yl)amino)acetate, 13

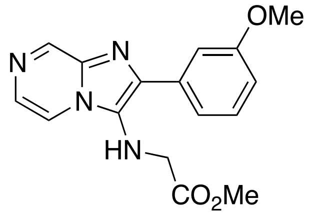

Yellow solid; mp = 137-138 ºC; 61%; ^{1}H NMR (CD_3OD, 400.13 MHz): δ 3.60 (3H, s, OCH_3), 3.88 (3H, s, OCH_3), 3.92 (2H, s, CH_2), 7.06 (1H, ddd, *J* = 8.6, 2.6 and 1.2 Hz, Ar-H), 7.45 (1H, t, *J* = 8.0 Hz, Ar-H), 7.53-7.60 (2H, m, Ar-H), 7.95 (1H, d, *J* = 5.4 Hz, Ar-H), 8.66 (1H, dd, *J* = 5.3 and 1.2 Hz, Ar-H), 9.05 (1H, d, *J* = 0.8 Hz, Ar-H). ^{13}C NMR (CD_3OD, 100.61 MHz): δ 48.21, 52.66, 55.90, 114.35, 116.45, 118.91, 121.18, 121.24, 124.15, 131.20, 133.94, 135.13, 137.42, 140.56, 161.60, 173.27. HRMS-ESI calc. ($C_{16}H_{16}N_4O_3+H^+$): 313.1295, found: 313.1294.

Methyl 2-((2-(3-fluorophenyl)imidazo[1,2-*a*]pyrazin-3-yl)amino)acetate, 14

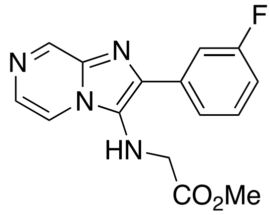

Yellow solid; mp = 170-171 ºC; 46%; ^{1}H NMR (CD_3OD, 400.13 MHz): δ 3.50 (3H, s, OCH_3), 3.87 (2H, s, CH_2), 7.12 (1H, tdd, *J* = 8.6, 2.4, 0.8 Hz, Ar-H), 7.46 (1H, m, Ar-H), 7.69 (1H, m, Ar-H), 7.77 (1H, m, Ar-H), 7.83 (1H, d, *J* = 5.2 Hz, Ar-H), 8.57 (1H, dd, *J* = 5.2 and 1.2 Hz, Ar-H), 8.97 (1H, d, *J* = 1.0 Hz, Ar-H). ^{13}C NMR (CD_3OD, 100.61 MHz): δ 48.41, 52.66, 115.41, 115.65, 117.11, 117.32, 119.29, 123.94, 124.72, 131.92, 135.44, 138.33, 163.28, 165.71, 173.31. HRMS-ESI calc. ($C_{15}H_{13}FN_4O_2+H^+$): 301.1095, found: 301.1097.

Methyl 2-((6-(2,4-dimethoxyphenyl)imidazo[2,1-*b*]thiazol-5-yl)amino)acetate, 15

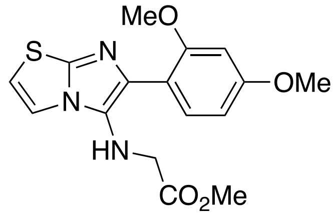

Yellow oil; 52%; ^{1}H NMR (CD_3OD, 400.13 MHz): δ 3.39 (3H, s, OCH_3), 3.58 (2H, s, CH_2), 3.68 (3H, s, OCH_3), 3.72 (3H, s, OCH_3), 6.46-6.53 (2H, m, Ar-H), 7.36 (1H, m, Ar-H), 7.46 (1H, m, Ar-H), 7.96 (1H, dd, *J* = 4.4 and 1.2 Hz, Ar-H). ^{13}C NMR (CD_3OD, 100.61 MHz): δ 48.78, 52.53, 56.12, 56.40, 99.75, 106.88, 109.20, 117.89, 121.03, 123.30, 130.74, 132.10, 143.16, 159.66, 164.09, 173.34. HRMS-ESI calc. ($C_{16}H_{17}N_3O_4S+H^+$): 348.1013, found: 348.1013.

HPLC chromatograms

For IDs for aminopyridines (P), aldehydes (A) and isocyanides (I), see page S5.

Compound {12, 5, 6}

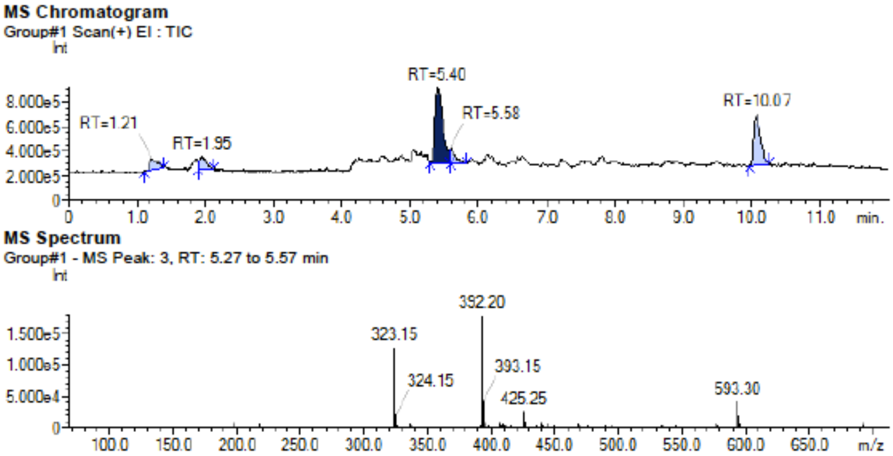

Compound {7, 5, 6}

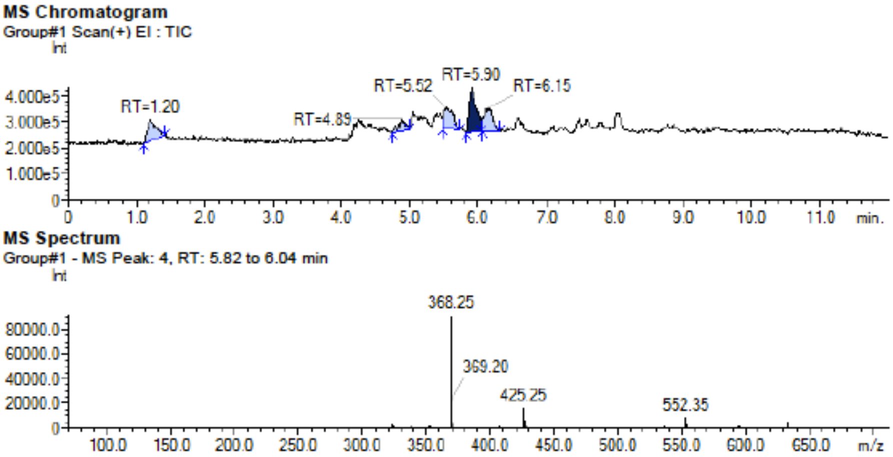

Compound {9, 5, 6}

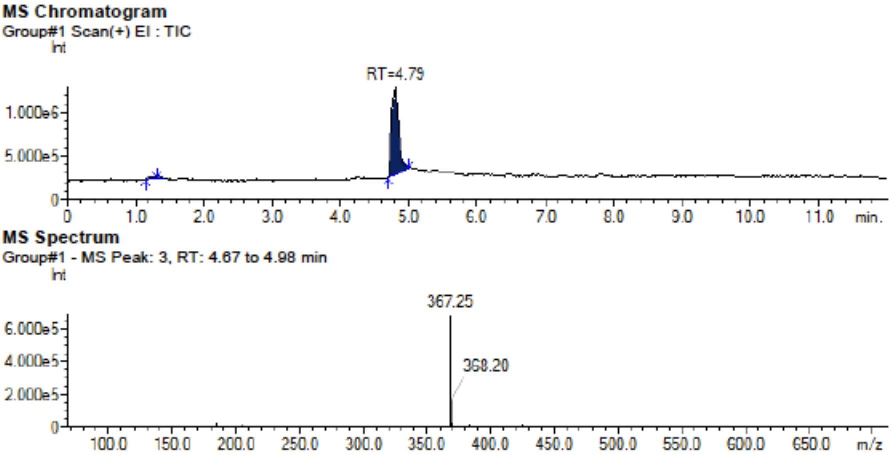

Compound {12, 5, 17}

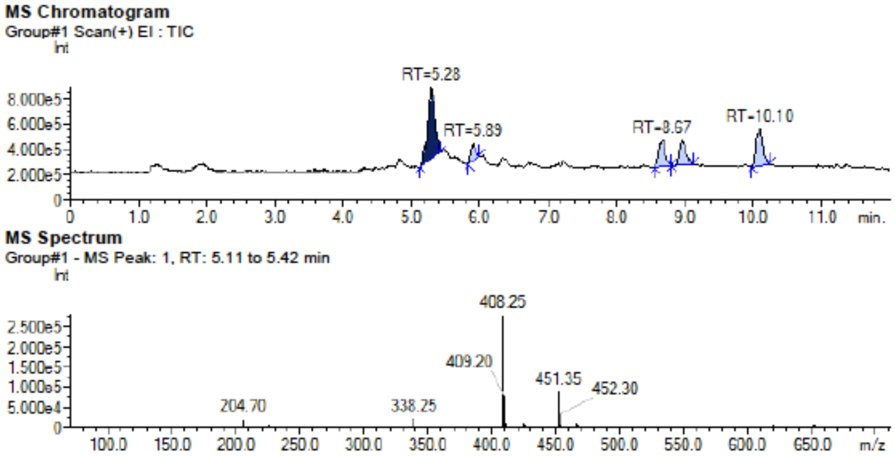

Compound {7, 5, 17}

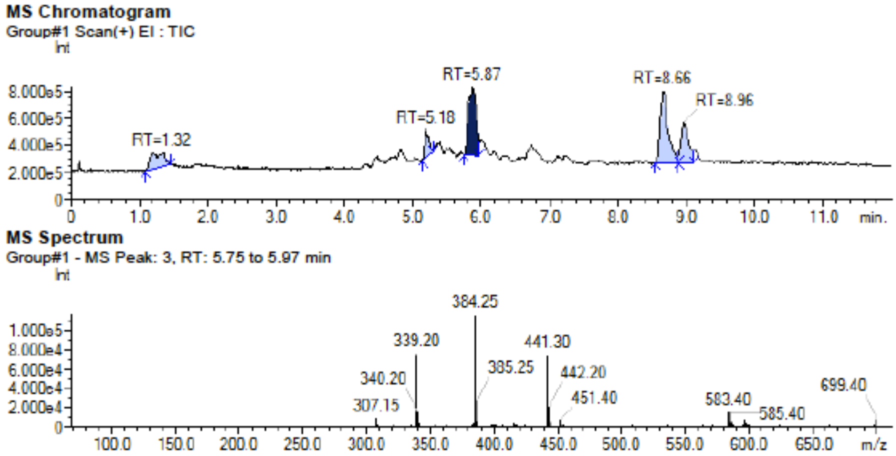

Compound {9, 5, 17}

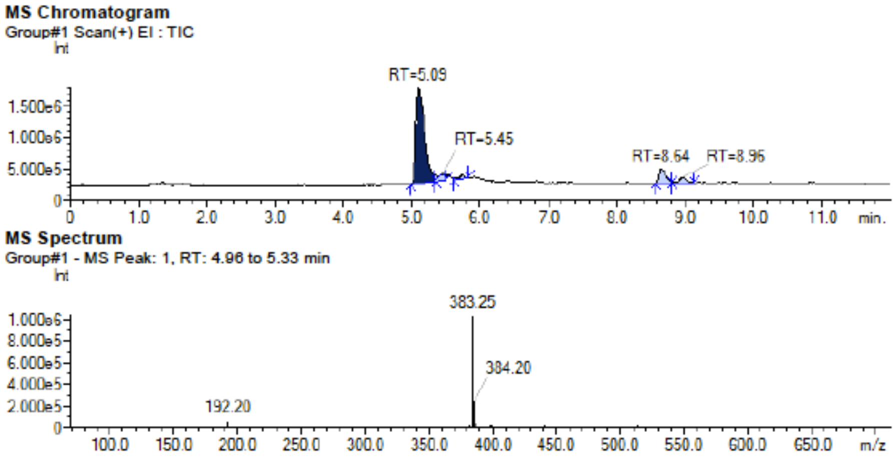

Compound {5, 5, 17}

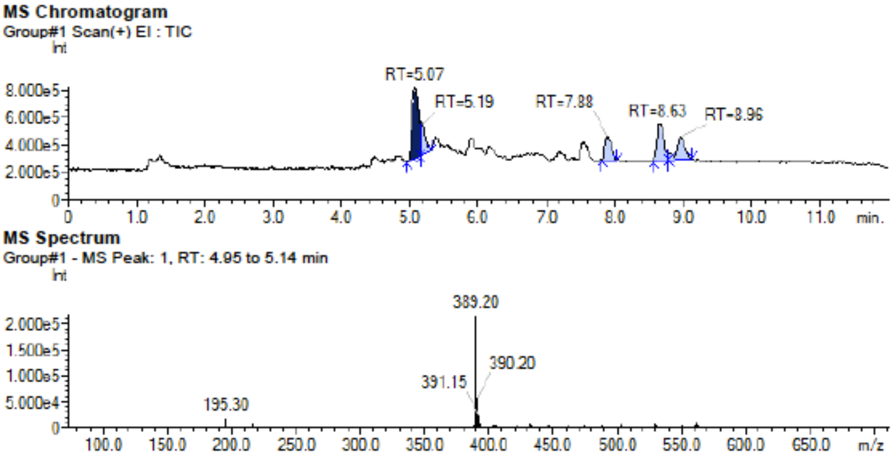

Compound {8, 5, 19}

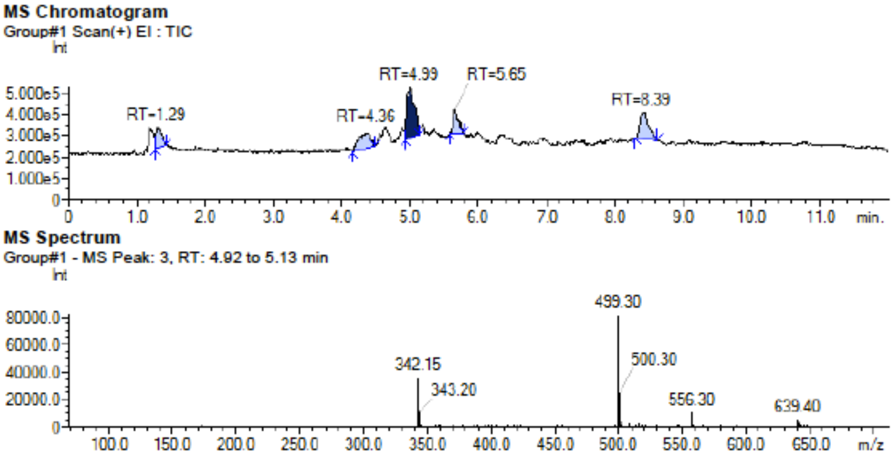

Compound {8, 5, 8}

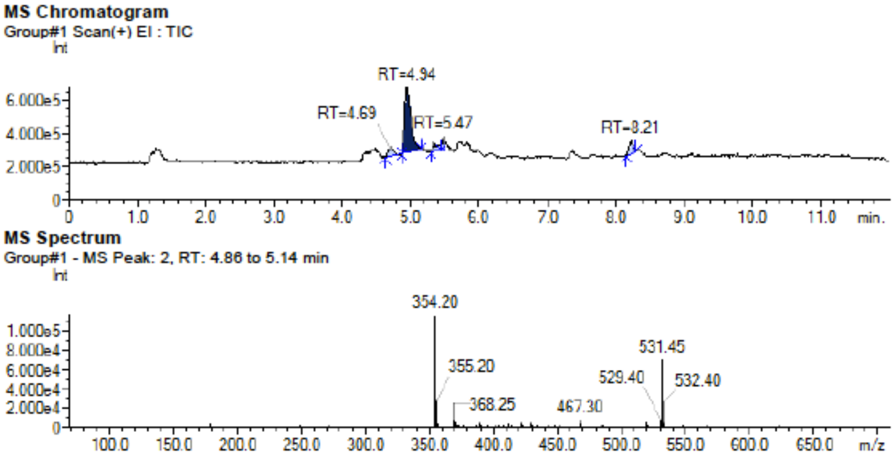

Compound {12, 5, 19}

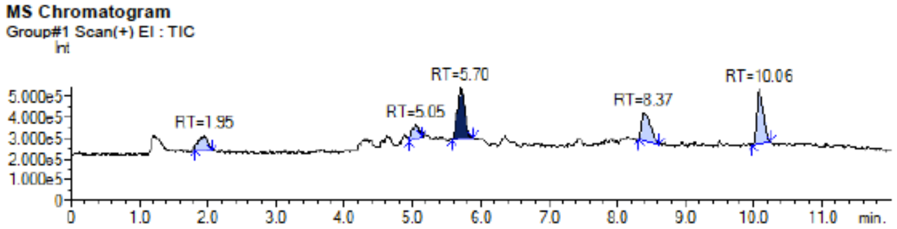

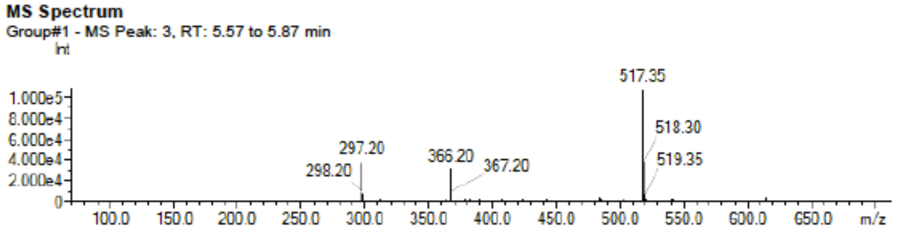

Compound {12, 5, 8}

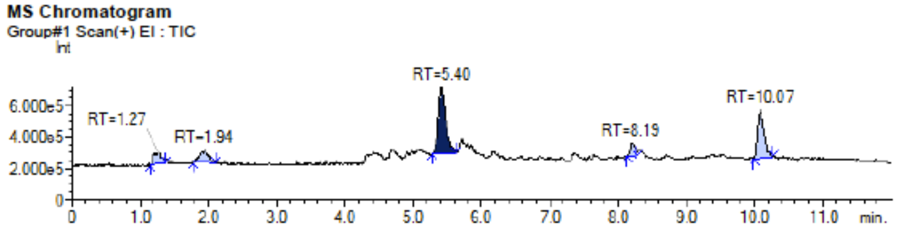

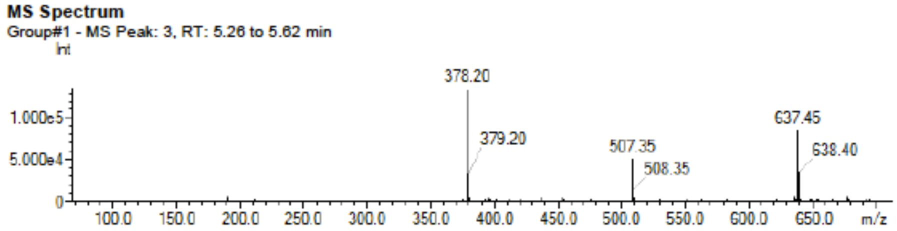

Compound {10, 5, 19}

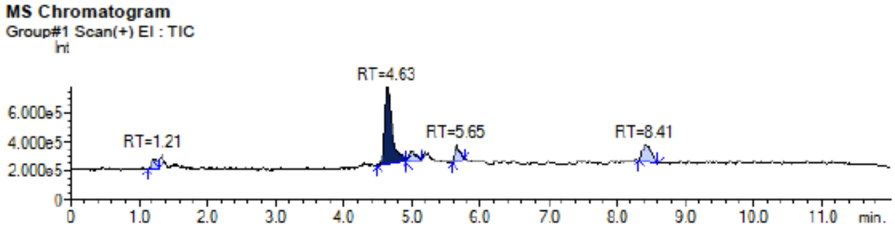

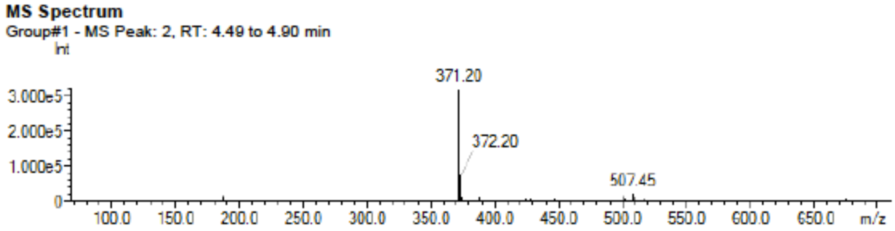

Compound {10, 5, 8}

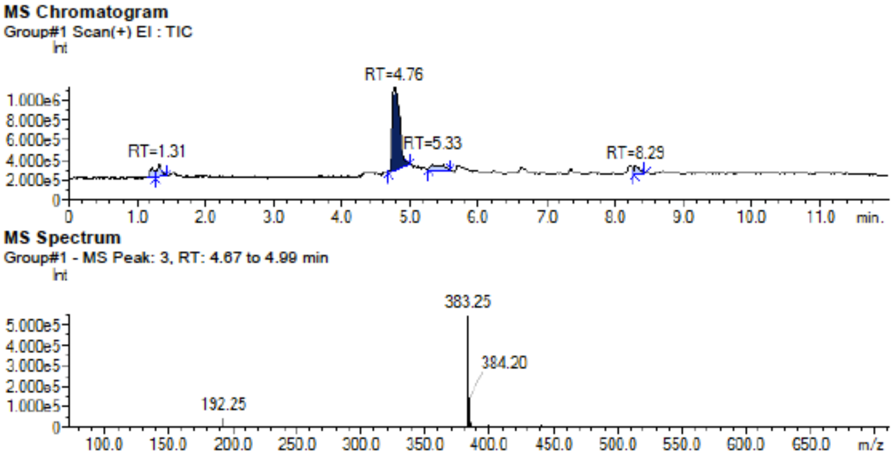

Compound {8, 5, 25}

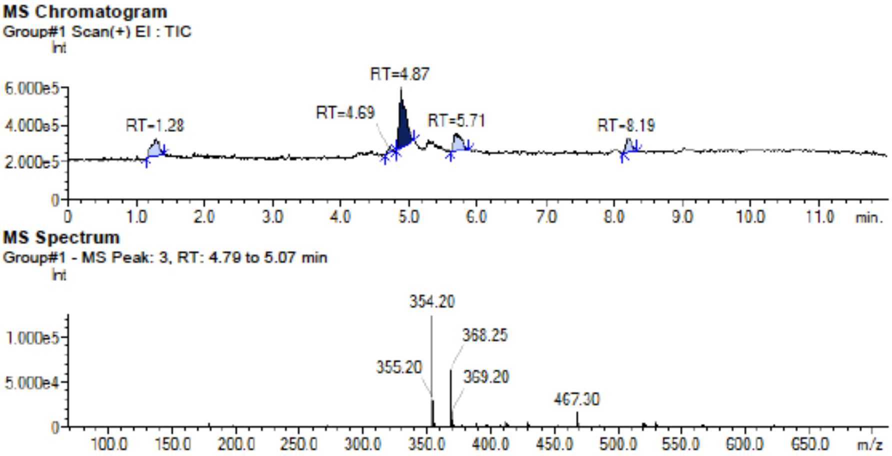

Compound {8, 5, 6}

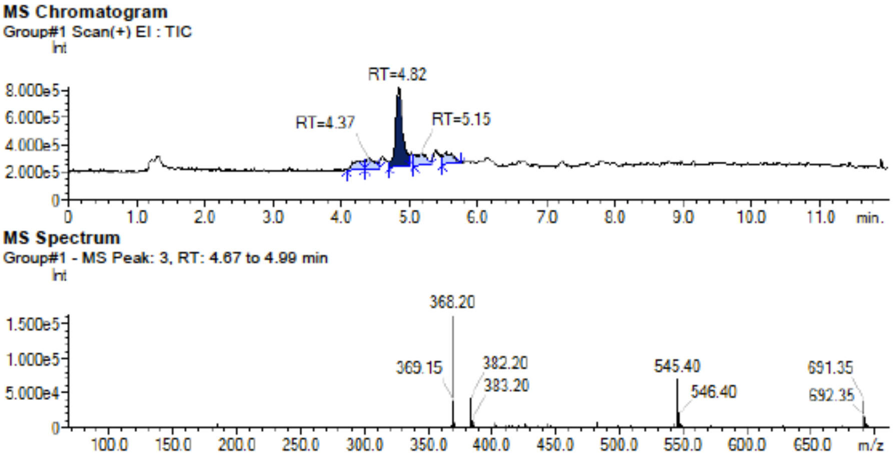

Compound {6, 5, 6}

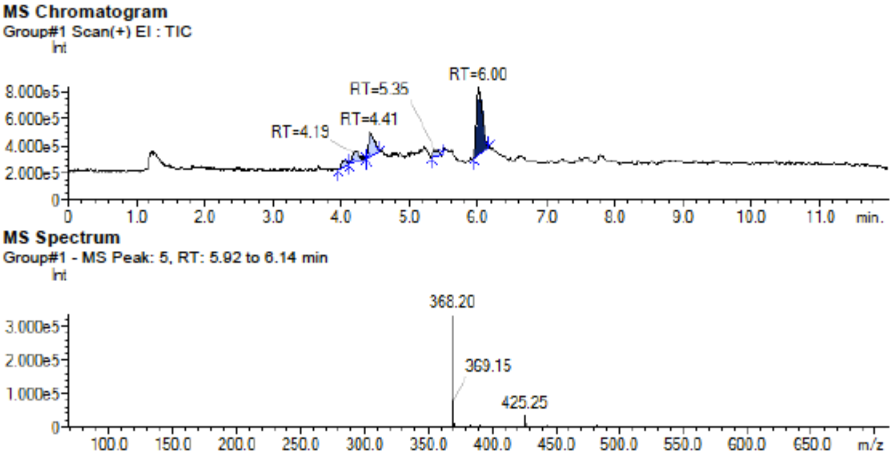

Compound {8, 5, 17}

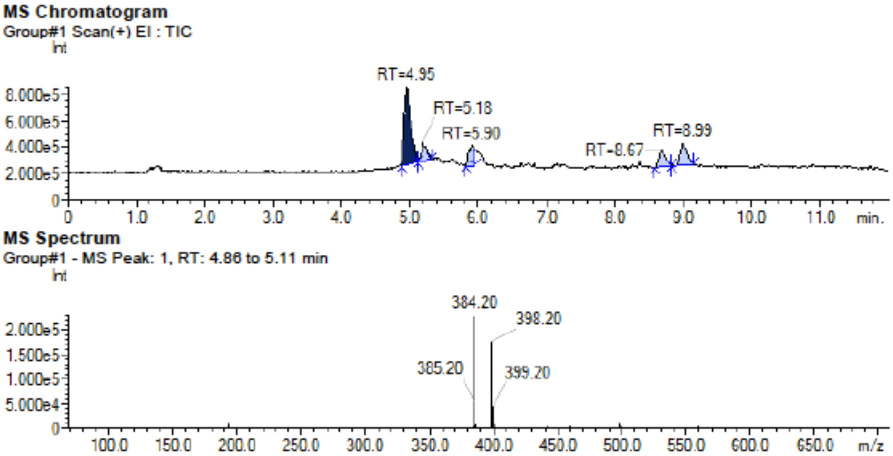

Compound {5, 5, 6}

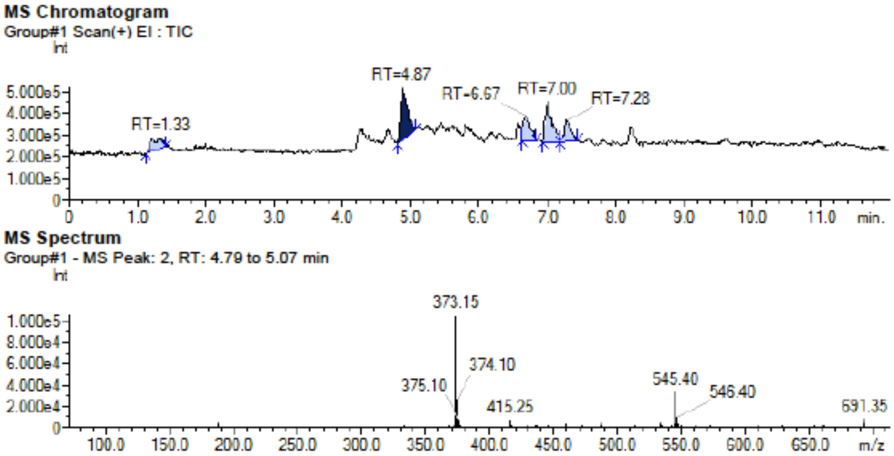

Compound {2, 5, 6}

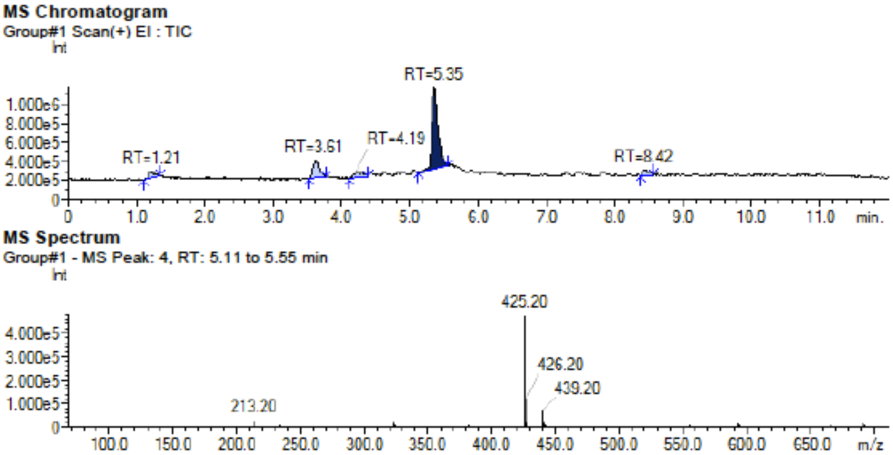

Compound {12, 5, 5}

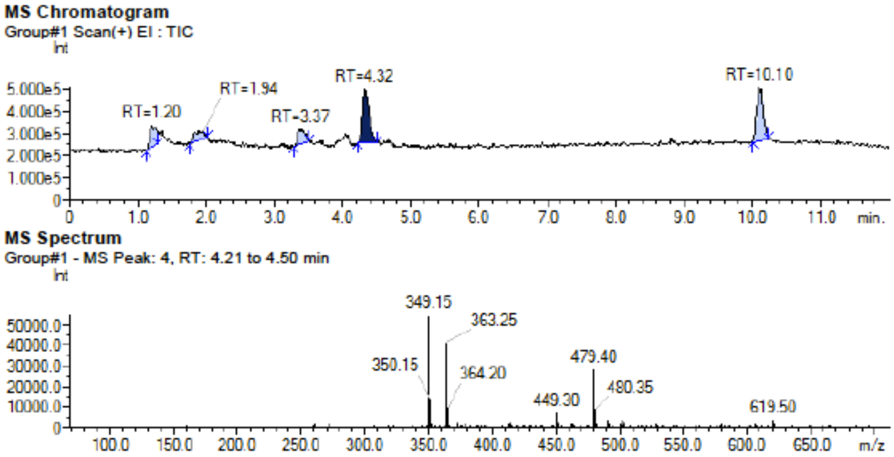

Compound {2, 5, 5}

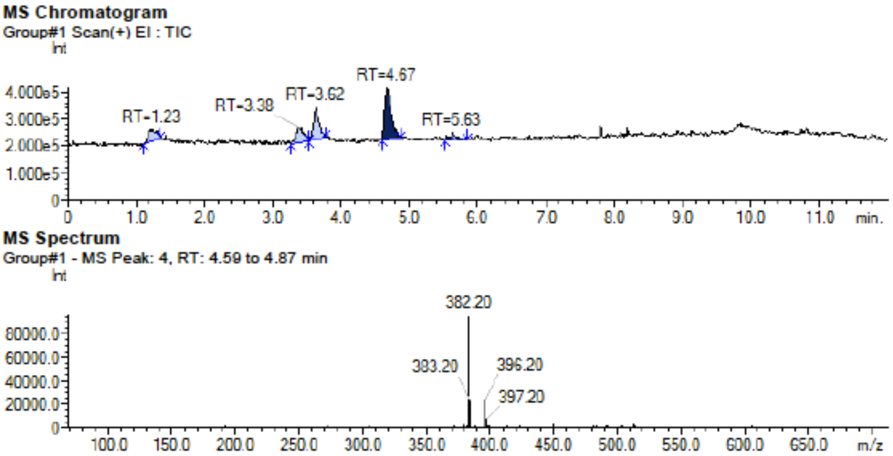

Compound {8, 5, 24}

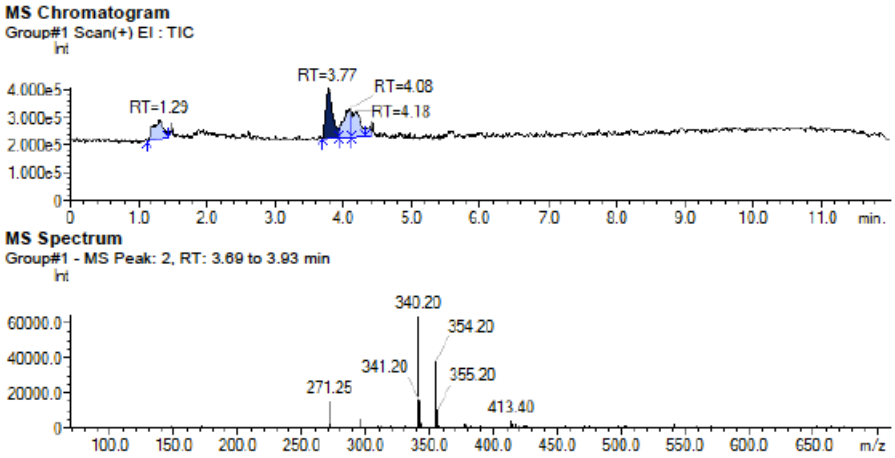

Compound {8, 5, 5}

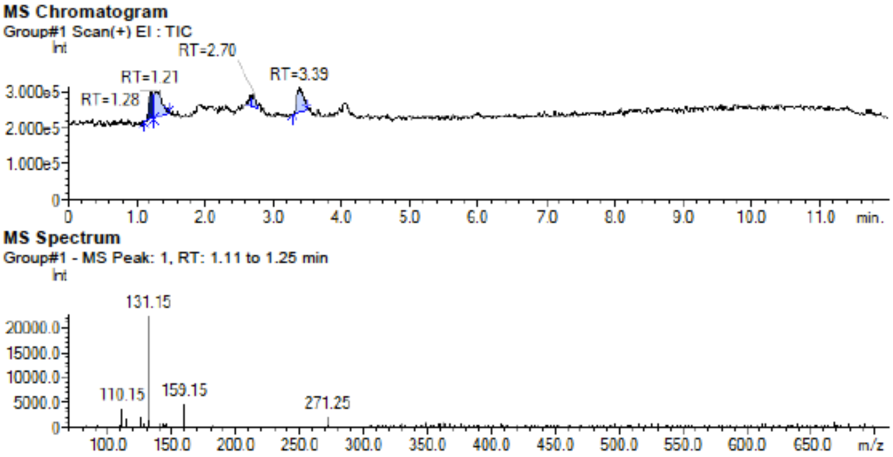

Compound {6, 5, 17}

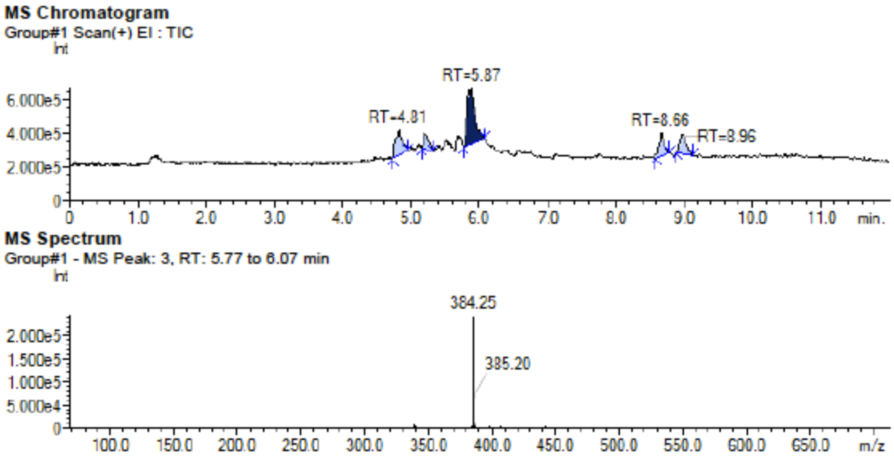

Compound {7, 5, 5}

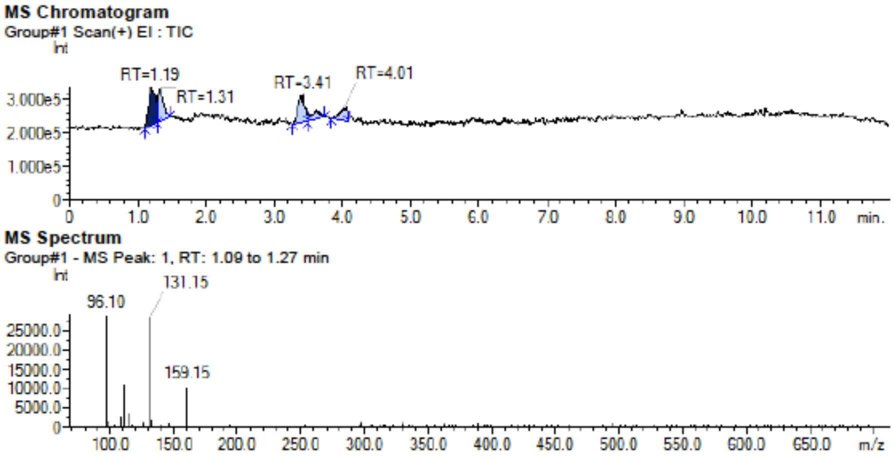

Compound {3, 5, 5}

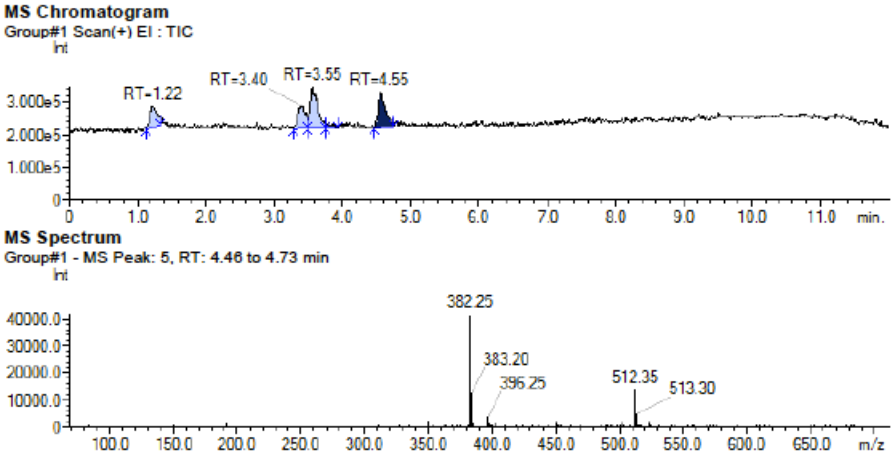

Compound {2, 5, 17}

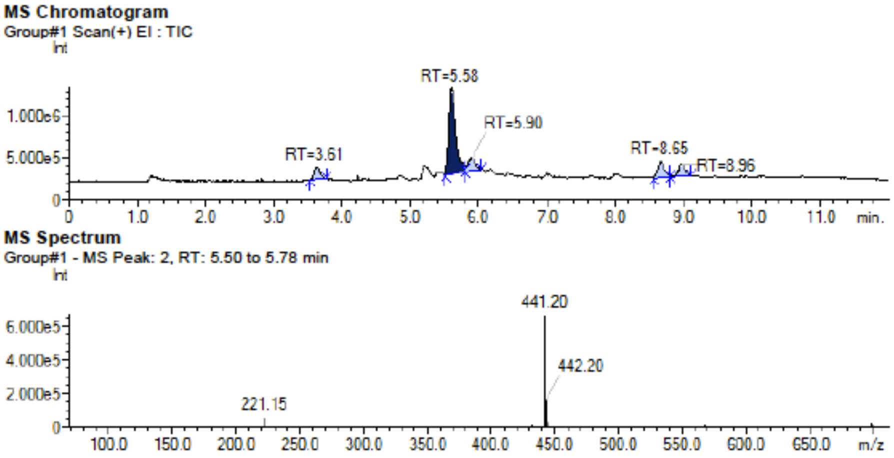

Compound {6, 5, 5}

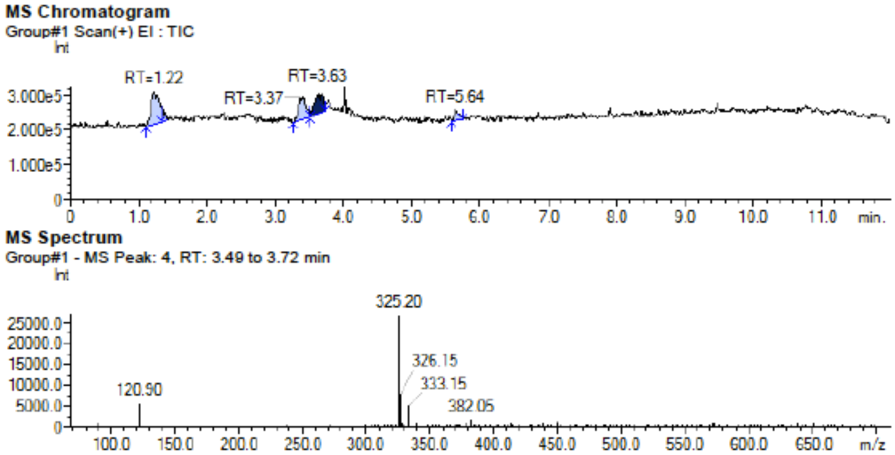

Compound {3, 5, 17}

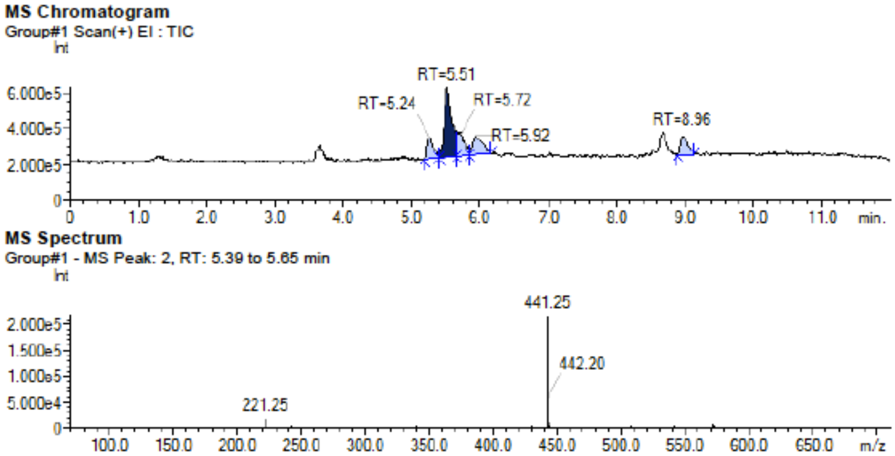

Compound {2, 5, 19}

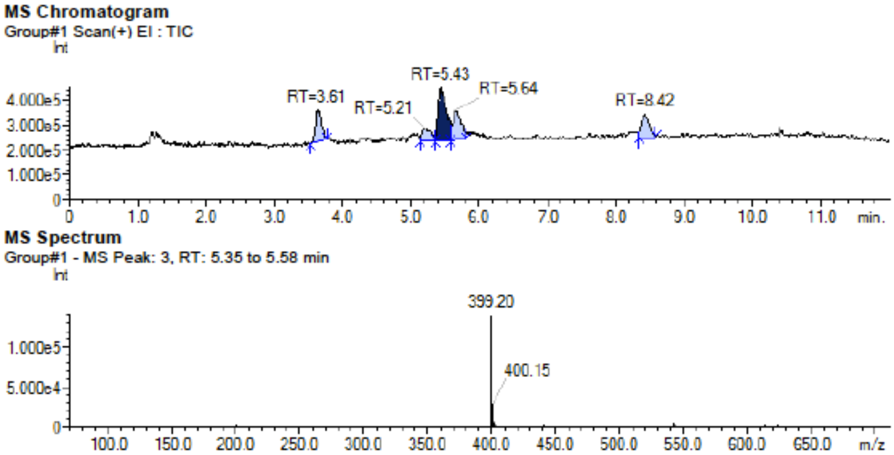

Compound {2, 5, 8}

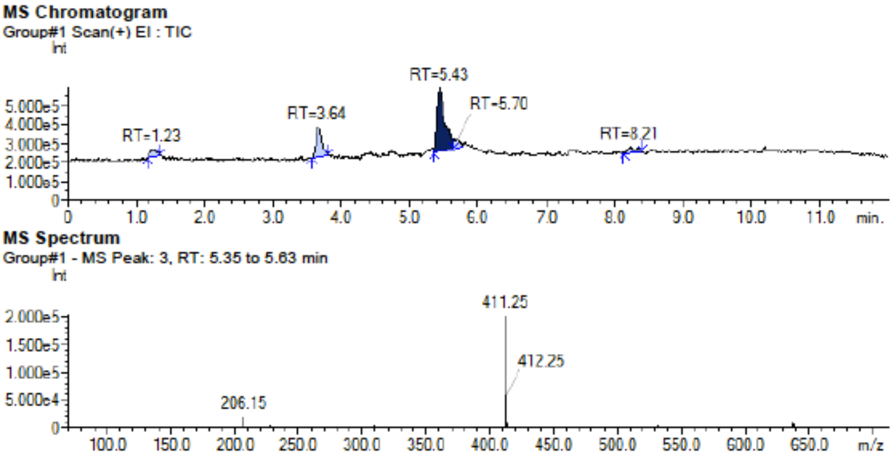

Compound {2, 5, 13}

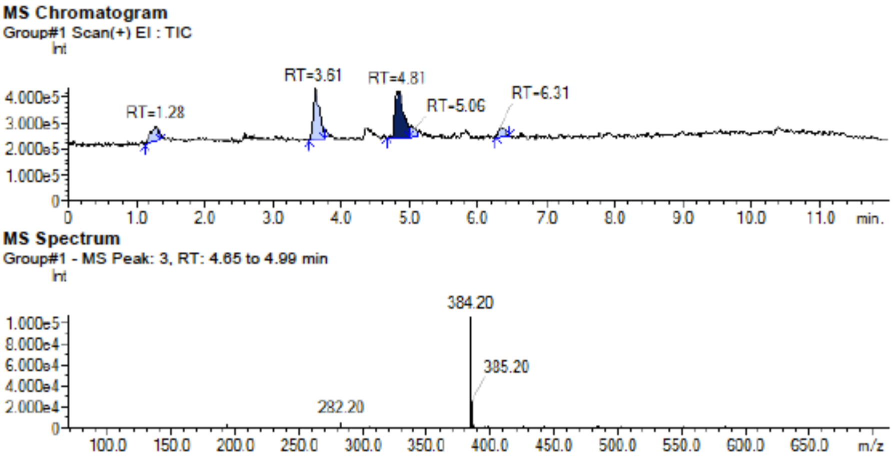

Compound {9, 5, 5}

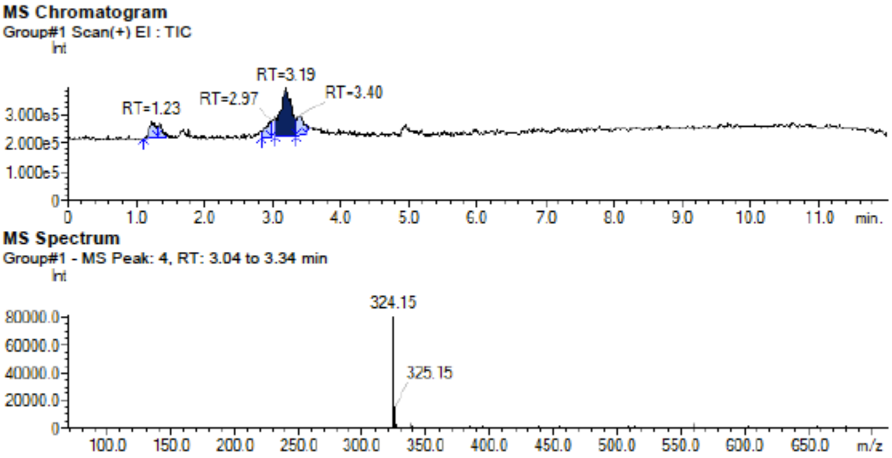

Compound {7, 5, 19}

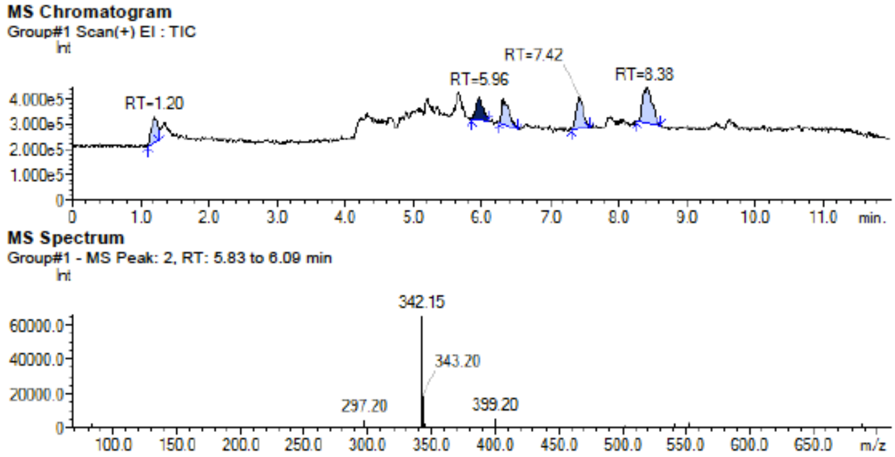

Compound {7, 5, 8}

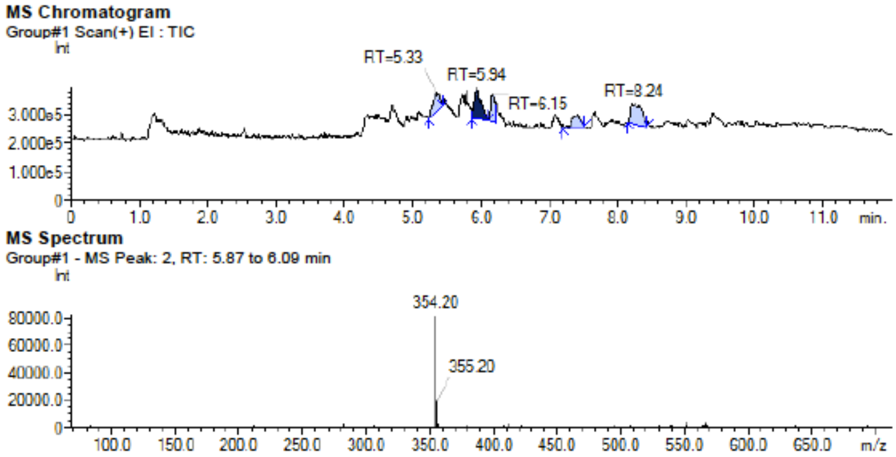

Compound {12, 5, 13}

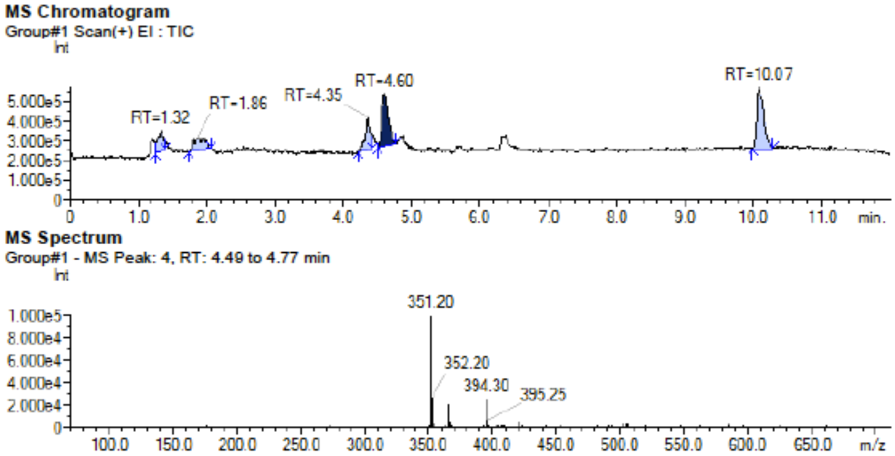

Compound {6, 5, 19}

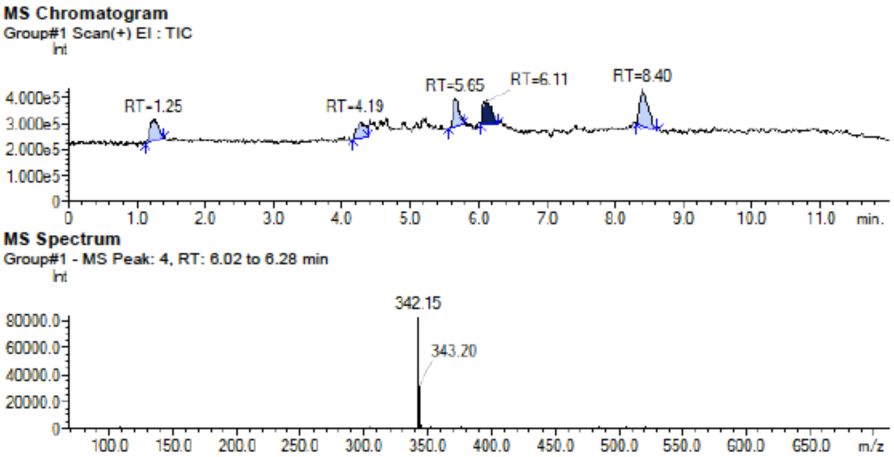

Compounds {6, 5, 8}

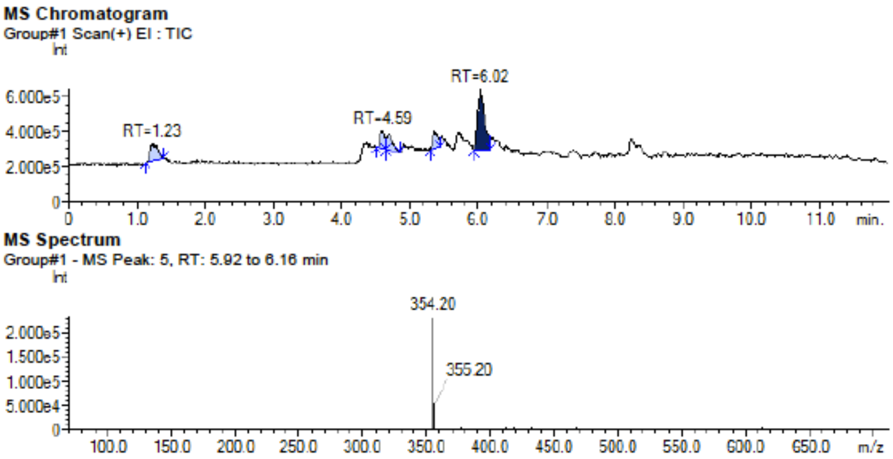

Compounds {1, 5, 25}

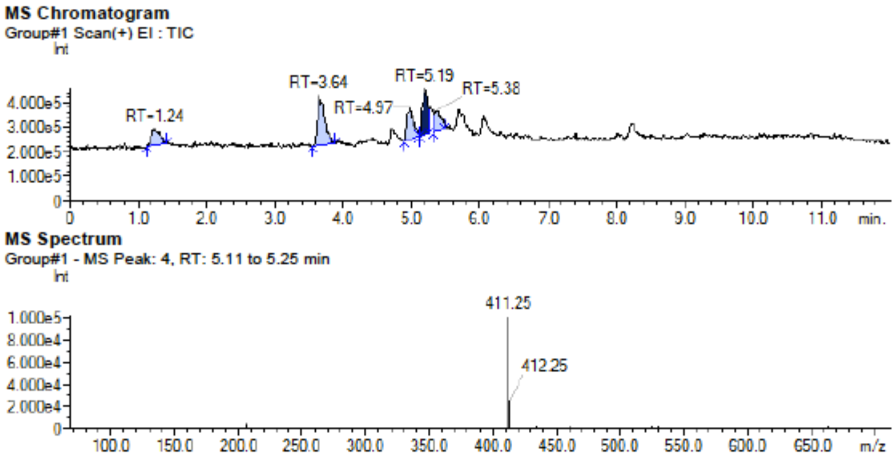

Compounds {8, 5, 27}

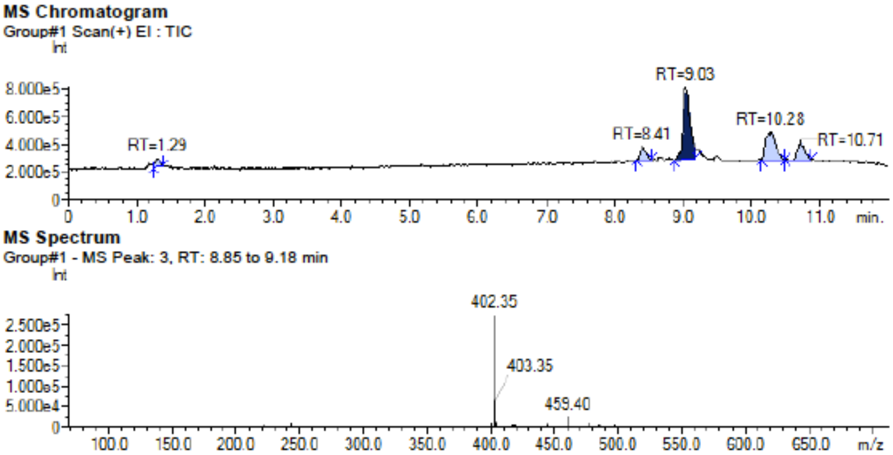

Compound {12, 5, 27}

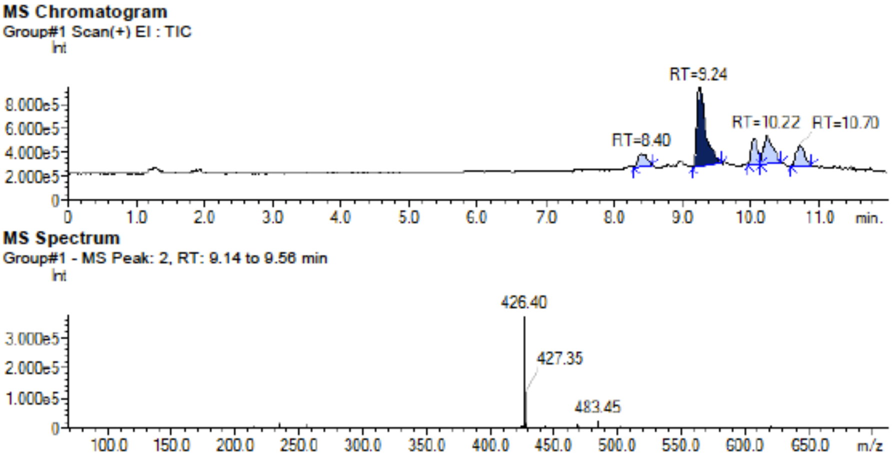

Compound {2, 5, 25}

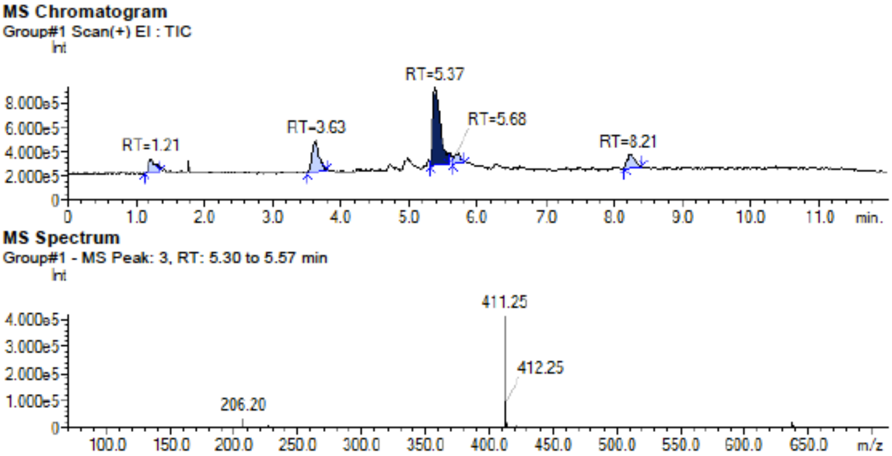

Compound {8, 5, 31}

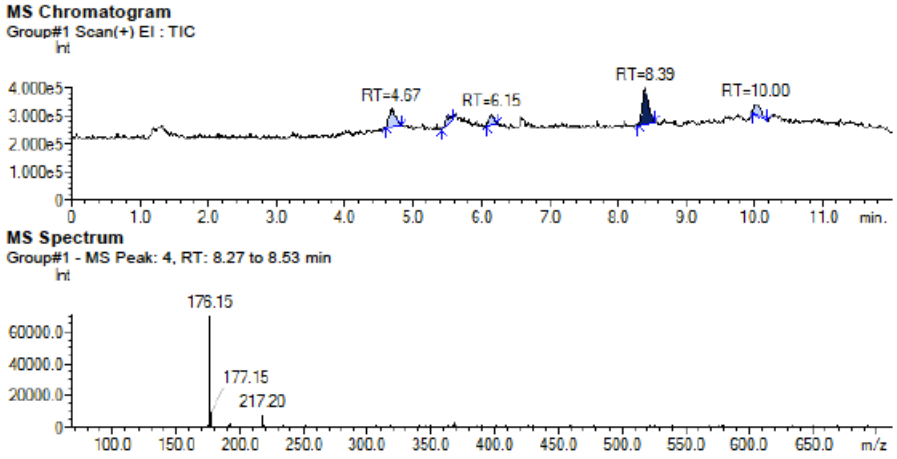

Compound {12, 5, 25}

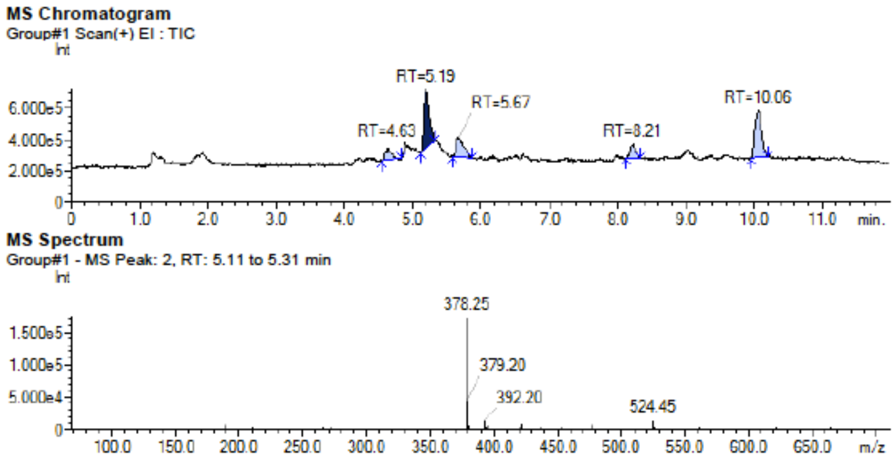

Compound {3, 5, 25}

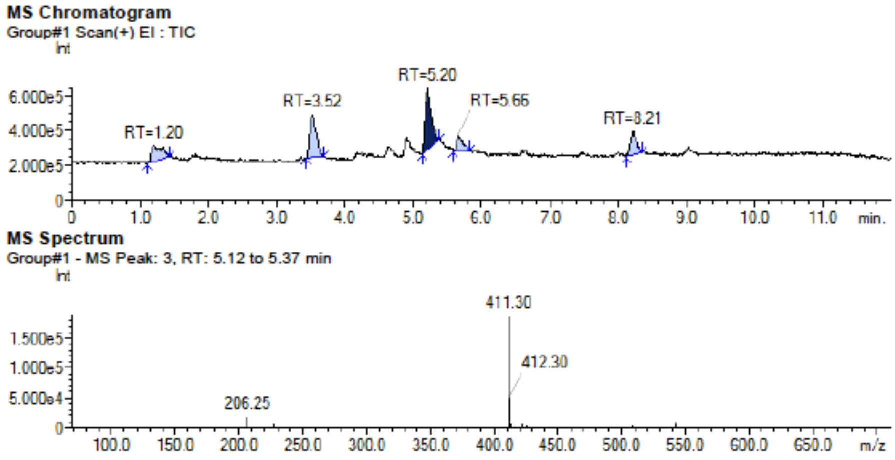

Compound {7, 5, 27}

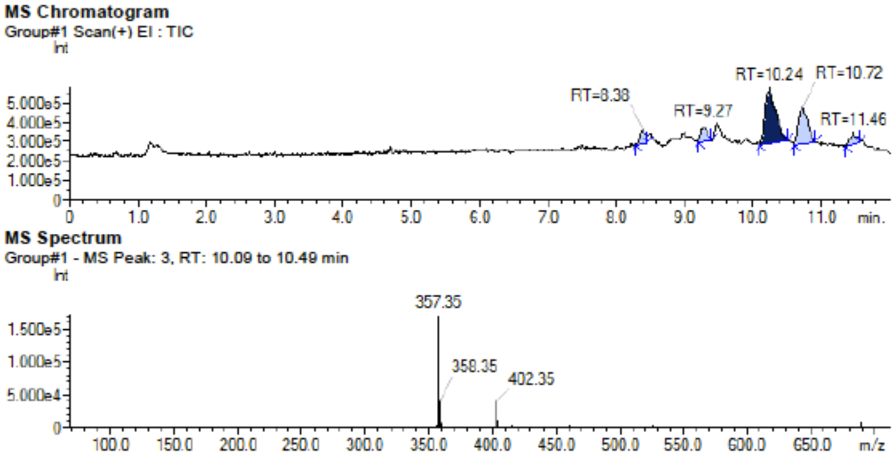

Compound {7, 5, 25}

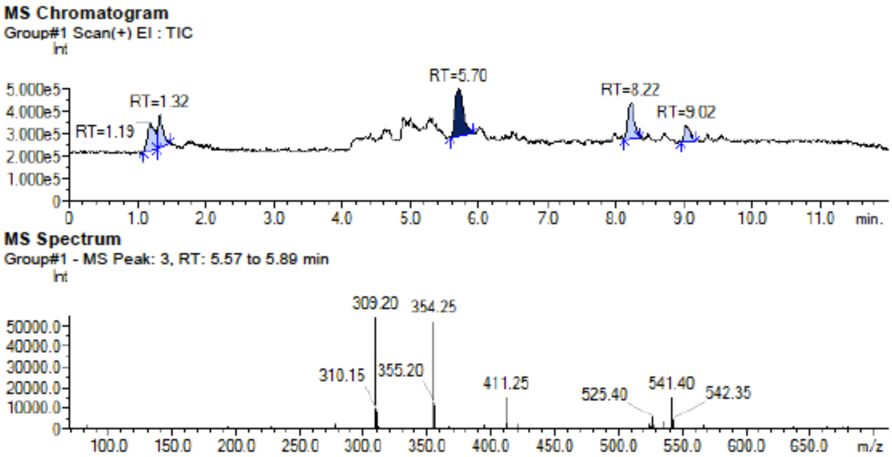

Compound {9, 5, 19}

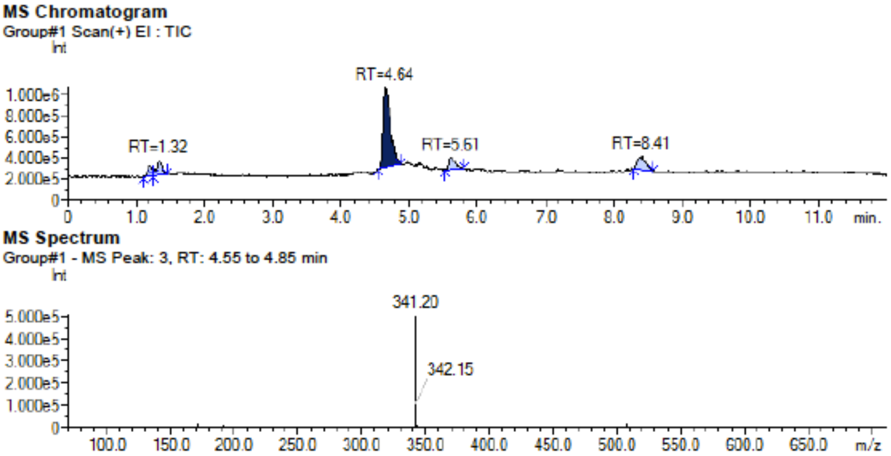

Compound {9, 5, 8}

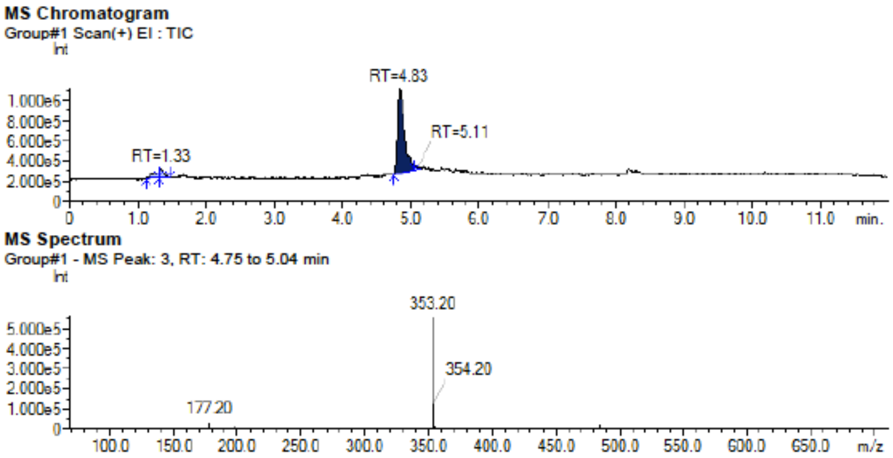

Compound {6, 5, 25}

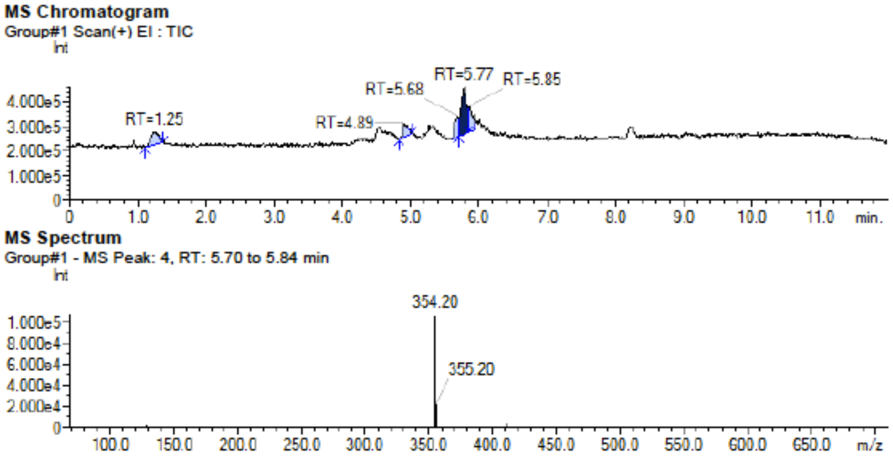

Compound {7, 5, 32}

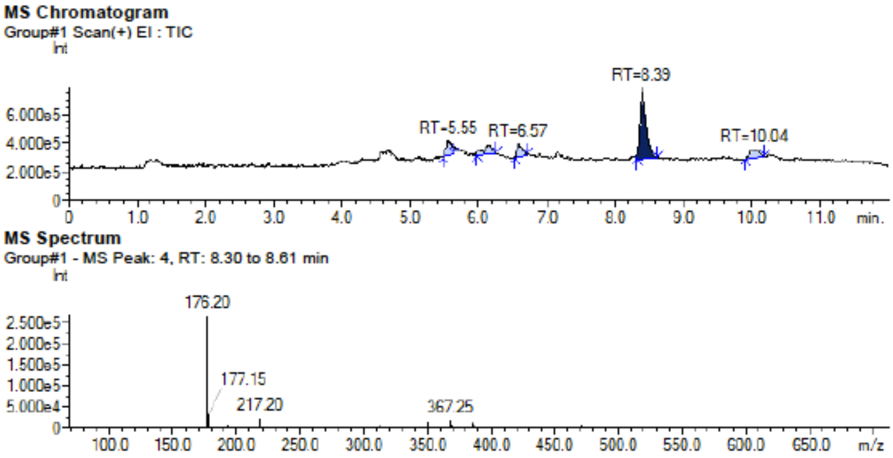

Compound {9, 5, 25}

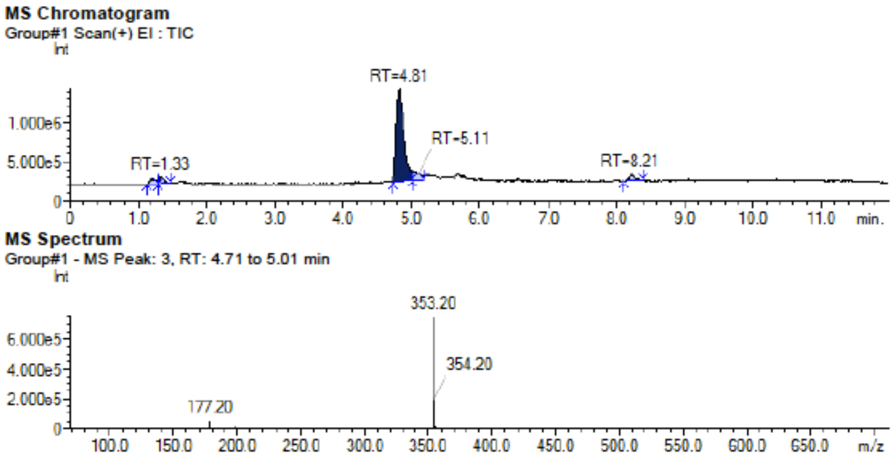

Compound {5, 5, 25}

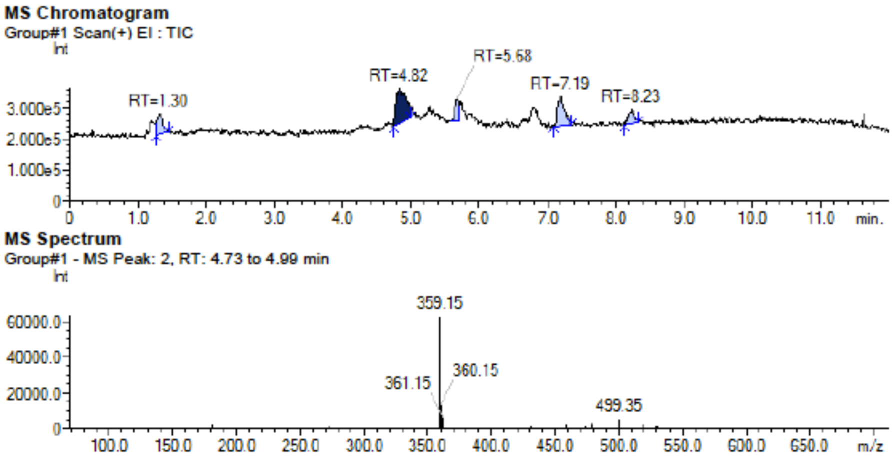

Compound {4, 5, 24}

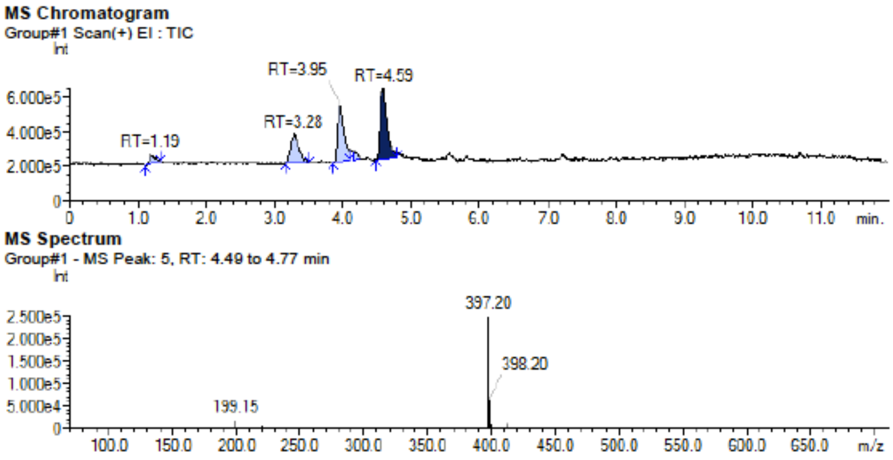

Compound {4, 5, 19}

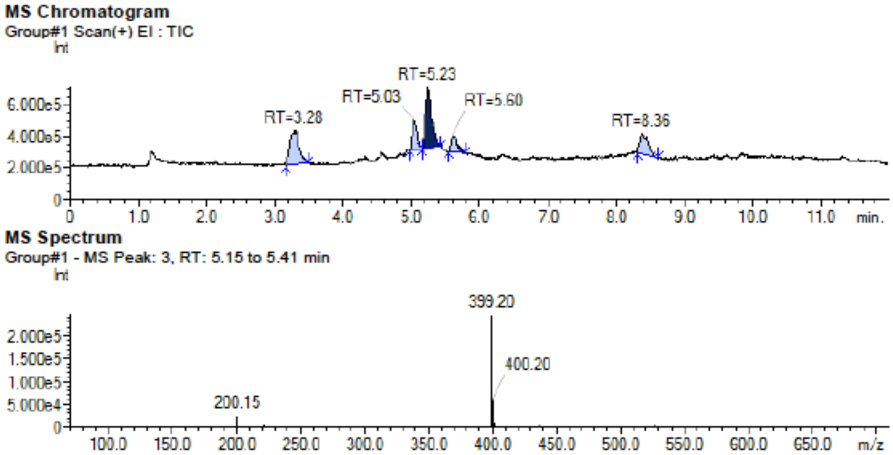

Compound {4, 5, 8}

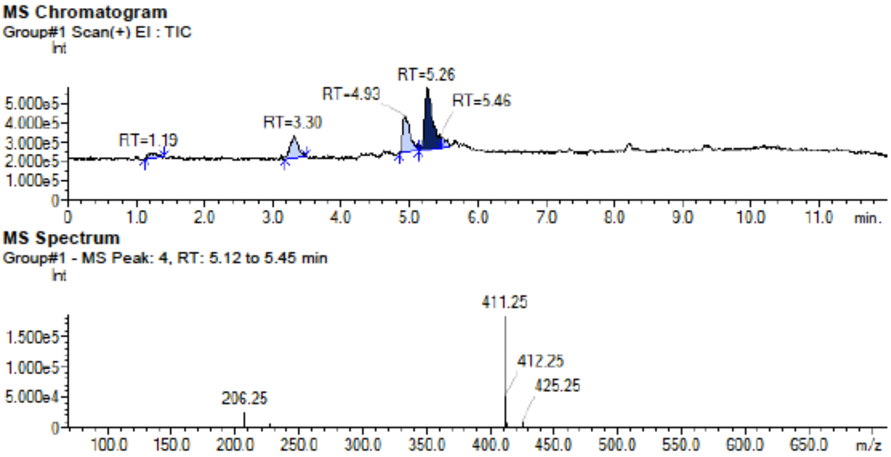

Compound {4, 5, 25}

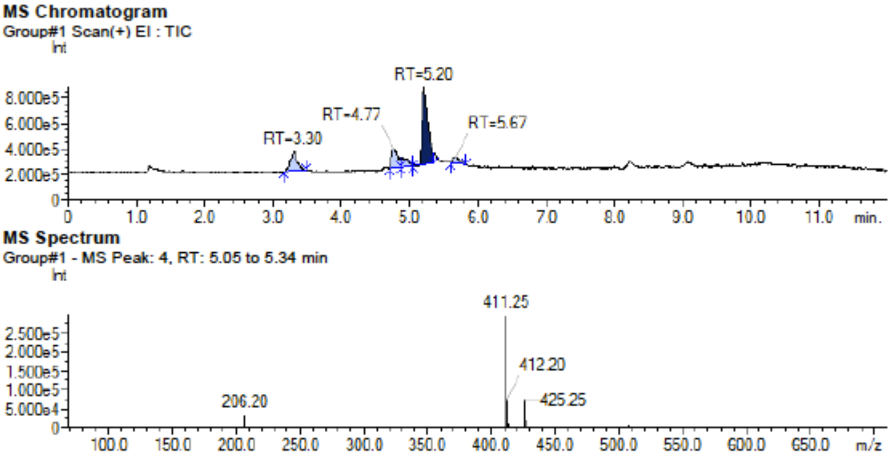

Preliminary screening data and IC_{50} values – PI3Kα

All compounds were tested in a single-dose duplicate at 75 μM. The control compound PI-103 was tested in 10-dose IC_{50} with 3-fold serial dilution starting at 10 μM. All reactions were carried out at 10 μM ATP.[3]

Table 1. Screening data for selected compounds.

**Entry**	**Compound (P, I, A)**	**Structure**	**Screening assay % Activity relative to DMSO**		**IC_{50} /μM**	**Compound ID in manuscript**
			**Measurement 1**	**Measurement 2**		
**1**	12, 5, 6	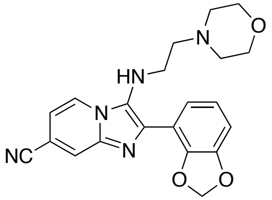	95.04	93.30	N.T.	
**2**	7, 5, 6	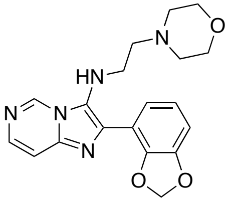	N.T.	N.T.	N.T.	
**3**	9, 5, 6	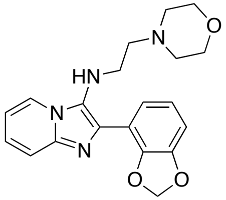	76.32	68.66	>600	**3**

**4**	12, 5, 17	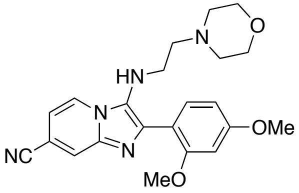	80.45	92.15	N.T.	
**5**	7, 5, 17	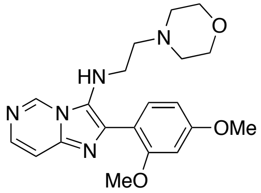	75.37	85.05	N.T.	
**6**	9, 5, 17	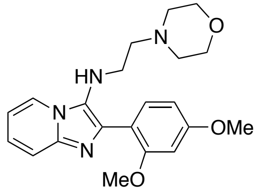	99.04	99.08	N.T.	
**7**	5, 5, 17	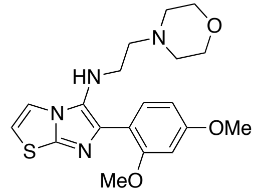	78.37	89.63	N.T.	
**8**	8, 5, 19	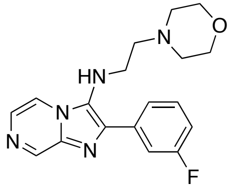	92.64	106.94	N.T.	

**9**	8, 5, 8	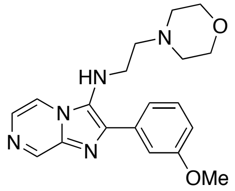	99.16	82.95	N.T.	
**10**	12, 5, 19	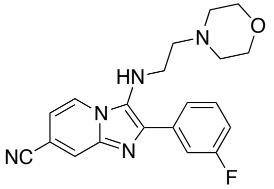	106.96	87.53	N.T.	
**11**	12, 5, 8	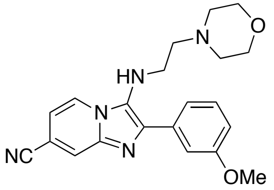	82.38	83.86	N.T.	
**12**	10, 5, 19	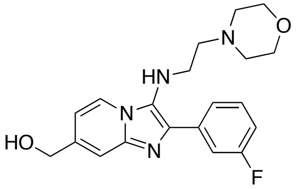	90.10	80.91	N.T.	
**13**	10, 5, 8	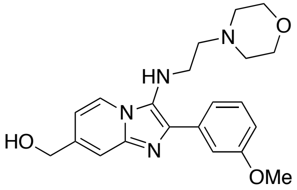	69.98	84.82	N.T.	

**14**	8, 5, 25	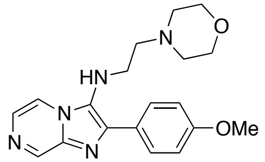	92.14	91.08	N.T.	
**15**	8, 5, 6	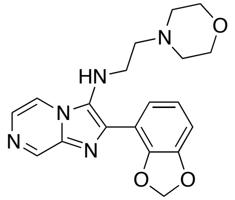	84.27	94.90	N.T.	
**16**	6, 5, 6	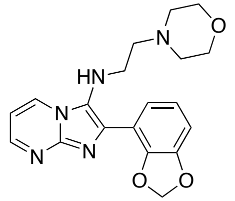	84.80	95.92	N.T.	
**17**	8, 5, 17	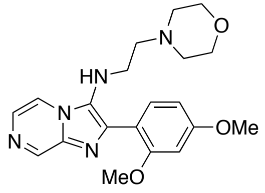	86.91	84.64	N.T.	
**18**	5, 5, 6	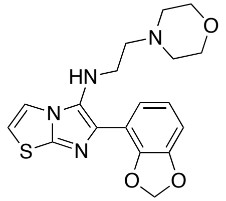	74.78	79.95	N.T.	

**19**	2, 5, 6	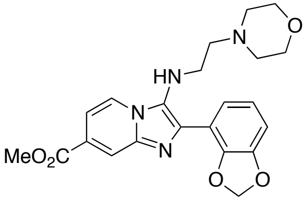	92.97	97.26	N.T.	
**20**	12, 5, 5	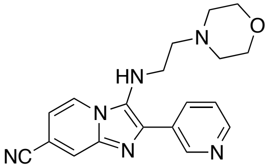	81.51	86.18	N.T.	
**21**	2, 5, 5	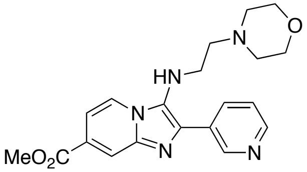	76.90	78.92	N.T.	
**22**	8, 5, 24	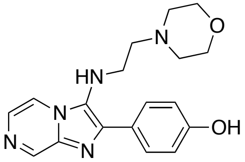	109.52	90.00	N.T.	
**23**	8, 5, 5	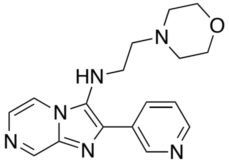	N.T.	N.T.	N.T.	
**24**	6, 5, 17	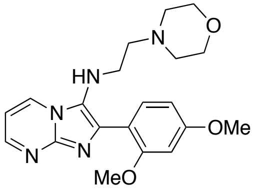	108.51	104.74	N.T.	

**25**	7, 5, 5	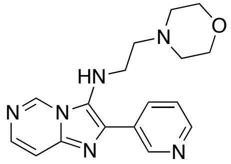	N.T.	N.T.	N.T.	
**26**	3, 5, 5	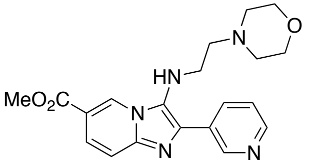	100.18	84.87	N.T.	
**27**	2, 5, 17	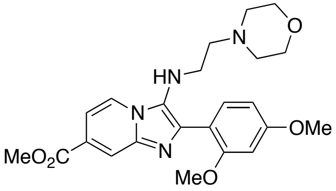	109.20	110.36	N.T.	
**28**	6, 5, 5	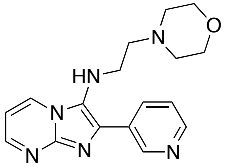	N.T.	N.T.	N.T.	
**29**	3, 5, 17	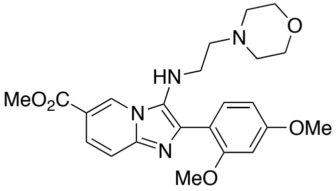	61.67	75.44	600	**4**
**30**	2, 5, 19	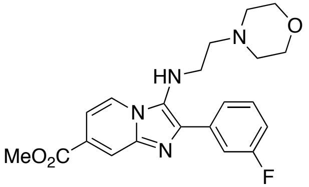	65.38	66.73	>600	**5**

**31**	2, 5, 8	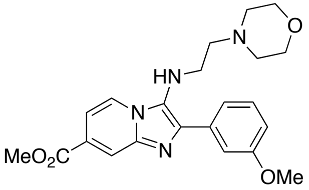	80.96	67.47	>600	**6**
**32**	2, 5, 13	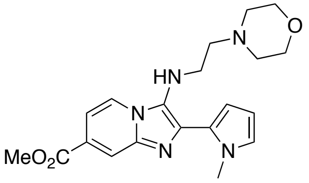	68.57	78.46	232	**2**
**33**	9, 5, 5	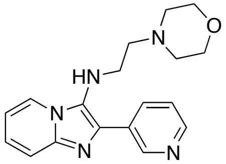	86.08	76.21	N.T.	
**34**	7, 5, 19	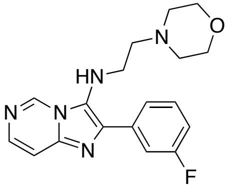	N.T.	N.T.	N.T.	
**35**	7, 5, 8	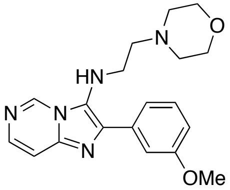	N.T.	N.T.	N.T.	

**36**	12, 5, 13	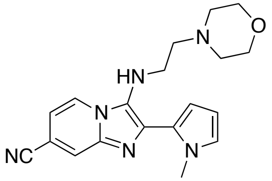	93.60	83.34	N.T.	
**37**	6, 5, 19	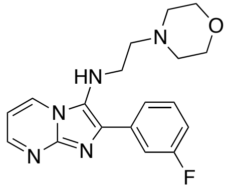	100.44	96.85	N.T.	
**38**	6, 5, 8	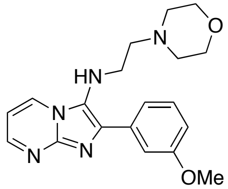	89.43	105.98	N.T.	
**39**	1, 5, 25	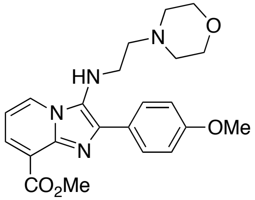	70.70	73.69	>600	**7**
**40**	8, 5, 27	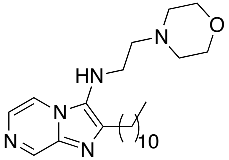	N.T.	N.T.	N.T.	

**41**	12, 5, 27	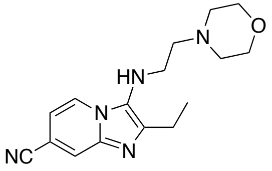	N.T.	N.T.	N.T.	
**42**	2, 5, 25	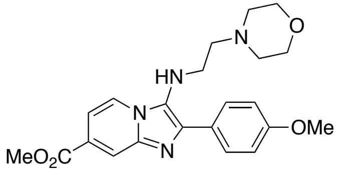	91.17	94.98	N.T.	
**43**	8, 5, 31	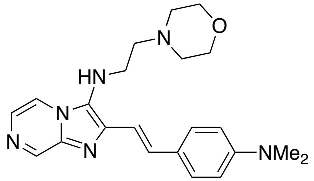	N.T.	N.T.	N.T.	
**44**	12, 5, 25	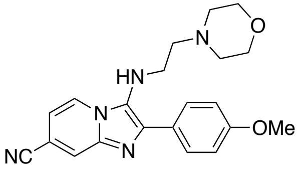	99.63	108.34	N.T.	
**45**	3, 5, 25	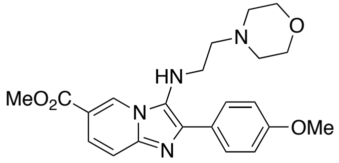	85.05	109.83	N.T.	
**46**	7, 5, 27	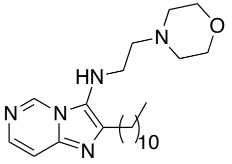	N.T.	N.T.	N.T.	

**47**	7, 5, 25	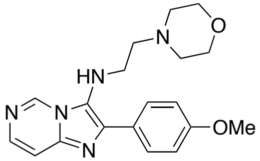	83.31	99.86	N.T.	
**48**	9, 5, 19	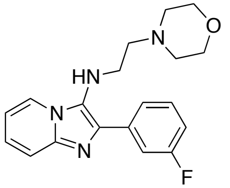	101.00	93.23	N.T.	
**49**	9, 5, 8	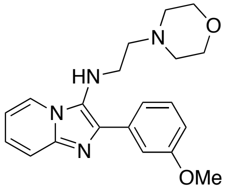	102.66	98.46	N.T.	
**50**	6, 5, 25	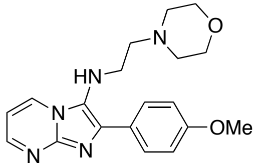	99.18	98.67	N.T.	
**51**	7, 5, 32	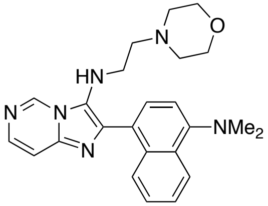	N.T.	N.T.	N.T.	

**52**	9, 5, 25	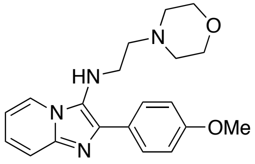	79.98	86.09	N.T.	
**53**	5, 5, 25	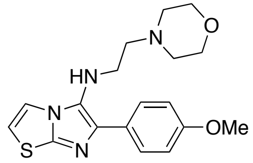	87.95	86.74	N.T.	
**54**	4, 5, 24	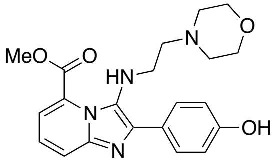	76.83	79.36	N.T.	
**55**	4, 5, 19	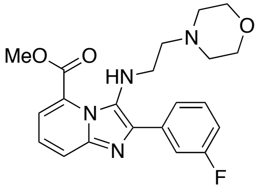	91.21	95.88	N.T.	
**56**	4, 5, 8	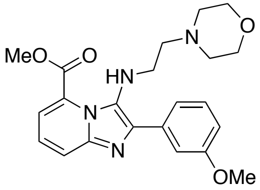	102.98	77.82	N.T.	
**57**	4, 5, 25	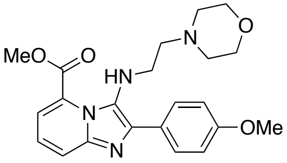	87.73	101.64	N.T.	

**58**	9, 4, 17	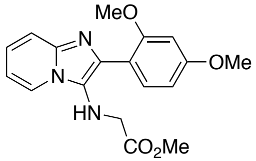	N.T.	N.T.	131	**1**
**59**	12, 4, 17	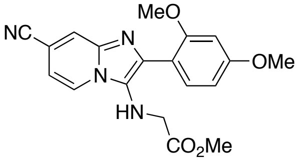	N.T.	N.T.	232	**8**
**60**	12, 4, 6	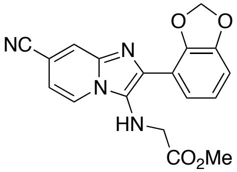	N.T.	N.T.	>600	**9**
**61**	8, 4, 25	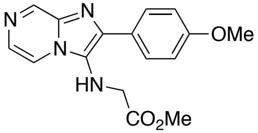	N.T.	N.T.	>600	**10**
**62**	12, 4, 5	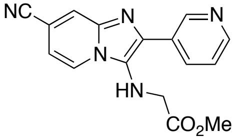	N.T.	N.T.	>600	**11**
**63**	9, 4, 6	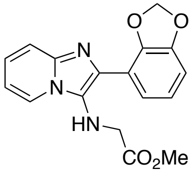	N.T.	N.T.	>600	**12**

**64**	8, 4, 8	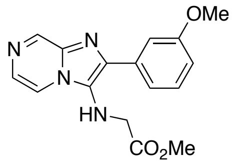	N.T.	N.T.	289	**13**
**65**	8, 4, 19	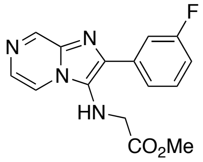	N.T.	N.T.	>600	**14**
**66**	5, 4, 17	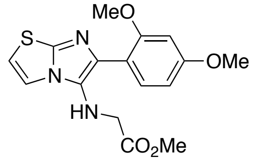	N.T.	N.T.	157	**15**

N.T. – Not tested; PI-103 presented an IC_{50} value of 4.05 nM under the assayed conditions.

References

1. Burchak, O. N.; Mugherli, L.; Ostuni, M.; Lacapère, J. J.; Balakirev, M. Y. *J. Am. Chem. Soc.* **2011**, *133*, 10058-10061.
2. Bienaymé, H.; Bouzid, K. *Angew. Chem. Int. Ed.* **1998**, *37*, 2234-2237.
3. Anastassiadis, T.; Deacon, S. W.; Devarajan, K.; Ma, H.; Peterson, R. J. *Nat. Biotechnol.* **2011**, *29*, 1039-1045.
